# Noble Metal‐Based Multimetallic Nanoparticles for Electrocatalytic Applications

**DOI:** 10.1002/advs.202104054

**Published:** 2021-11-17

**Authors:** Hyunjoong Kim, Tae Yong Yoo, Megalamane S. Bootharaju, Jeong Hyun Kim, Dong Young Chung, Taeghwan Hyeon

**Affiliations:** ^1^ Center for Nanoparticle Research Institute for Basic Science (IBS) Seoul 08826 Republic of Korea; ^2^ School of Chemical and Biological Engineering and Institute of Chemical Processes Seoul National University Seoul 08826 Republic of Korea; ^3^ Department of Chemistry Gwangju Institute of Science and Technology (GIST) Gwangju 61005 Republic of Korea

**Keywords:** electrocatalysis, multimetallic nanoparticles, nanoparticle synthesis, noble metal

## Abstract

Noble metal‐based multimetallic nanoparticles (NMMNs) have attracted great attention for their multifunctional and synergistic effects, which offer numerous catalytic applications. Combined experimental and theoretical studies have enabled formulation of various design principles for tuning the electrocatalytic performance through controlling size, composition, morphology, and crystal structure of the nanoparticles. Despite significant advancements in the field, the chemical synthesis of NMMNs with ideal characteristics for catalysis, including high activity, stability, product‐selectivity, and scalability is still challenging. This review provides an overview on structure‐based classification and the general synthesis of NMMN electrocatalysts. Furthermore, postsynthetic treatments, such as the removal of surfactants to optimize the activity, and utilization of NMMNs onto suitable support for practical electrocatalytic applications are highlighted. In the end, future direction and challenges associated with the electrocatalysis of NMMNs are covered.

## Graphical abstract

Intriguing surface and structural properties makes noble metal‐based multimetallic nanoparticles (NMMNs) efficient catalysts for a wide‐range of electrocatalytic applications. This review provides an overview of the general preparation of electrocatalysts using NMMNs, including synthesis of NMMNs with precise control, and practical issues for applying the nanoparticles to electrocatalytic reactions.

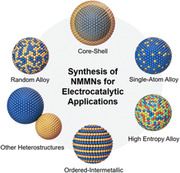

## 1 Introduction

Electrocatalytic reactions typically comprise adsorption and desorption of the involved molecular species on the surface of the catalysts. The Sabatier principle, which correlates the binding strength of reaction intermediates on active sites with catalytic activity, suggests that the interaction should neither be too strong nor too weak.^[^ ^1^ ^]^ It is widely accepted that noble metals (mainly Pt, Pd, Ir, Rh, Ru, and Au) have great potential as electrocatalysts due to their optimal sorption properties in volcano‐shaped activity trends for many representative electrocatalytic reactions that comprise fuel cells, water splitting, and CO_2_ electrolysis.^[^ ^2^, ^3^ ^]^ These metals are usually utilized in various forms of nanoparticles to take advantage of their maximized surface area and defect‐rich surfaces. The surface properties are well known to strongly influence the overall catalytic performance of noble metal nanoparticles.^[^ ^4^, ^5^ ^]^ However, monometallic systems are suffering from an intrinsically limited activity enhancement as well as instability under operating conditions.^[^ ^6^ ^]^ Furthermore, scarcity and high demand together made noble metals not affordable. Therefore, noble metal‐based monometallic catalysts are considered less promising for industrial applications, which typically require excellent activity, chemical and electrochemical stability, and cost‐effectiveness.

Over the past few decades, noble metal‐based multimetallic nanoparticles (NMMNs) synthesized by incorporating secondary metal elements into noble metal systems have emerged as alternatives. These nanoparticles have garnered tremendous attention because of their superior catalytic activity and durability compared to their monometallic counterparts.^[^ ^7^, ^8^ ^]^ In addition to the improvement in the overall catalytic performance, the adoption of non‐noble metals reduces the load on expensive noble metals, enabling economically feasible processes for manufacturing nanocatalysts. Pt‐based multimetallic nanoparticles are representative examples of enhanced catalytic activity and durability for the oxygen reduction reaction (ORR) compared to commercial Pt catalysts.^[^ ^9^, ^10^ ^]^ With the growing demand for electrocatalysts with superior performance, design principles have been more sophisticated by both empirical and theoretical methodologies to meet the need for multifunctionality. As shown in the previous studies, the physicochemical properties and catalytic performance of NMMNs are largely affected by the atomic distribution, size, composition, and morphology.^[^ ^11^, ^12^, ^13^, ^14^ ^]^ However, synthesizing NMMNs as per the design is challenging because the mechanism of the chemical synthesis of nanoparticles is complex and it is largely unclear. Nevertheless, more advanced and complicated synthetic procedures have been extensively developed to precisely modulate the structural factors, thereby achieving the desired properties of NMMNs.

Although there have been great achievements in the synthesis of nanoparticles, the as‐prepared nanoparticles themselves are not efficient electrocatalysts, leaving several more factors to be considered to successfully translate them into practical electrocatalysts. In the general synthesis of nanoparticles, surface capping agents (ligands) are prerequisite in achieving uniform size and controlled shapes with excellent dispersion. As the ligands can either have (usually) detrimental or (rarely) promotive effects on electrocatalytic processes, it is essential to consider them for better utilization of NMMNs.^[^ ^3^, ^15^ ^]^ On the other hand, catalytically active nanoparticles are usually anchored on the surface of support materials before being applied in catalytic reactions to prevent the agglomeration of nanoparticles and also to ensure sufficient electrical conductivity.^[^ ^16^ ^]^ Besides, the support materials can also affect the catalytic properties of nanoparticles through metal‐support interactions,^[^ ^17^ ^]^ and the stability of supports becomes significant, especially when the operating conditions are harsh or long‐term operation is required.^[^ ^3^ ^]^

**Figure 1:**
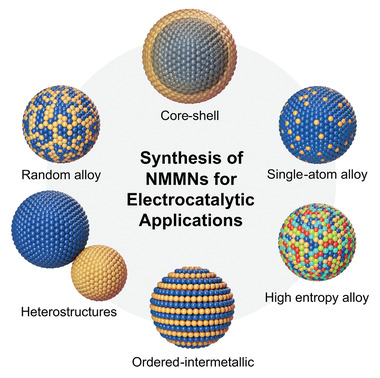
Categorization of NMMNs for electrocatalytic applications based on their structural characteristics.

In this review, recent developments in the synthesis of NMMNs, which are categorized based on their structural features (**Figure** **1**), are highlighted. For each structure, representative synthetic methods with exemplary works are provided. Along with the notable early contributions, major current synthetic challenges and some of the recent attempts to tackle them are discussed. Afterward, postsynthetic treatments/modifications that need to be considered to deal with the practical electrocatalytic applications of NMMNs, such as the removal of ligands and selection of the supports are covered.

## 2 Synthetic Methods for Various NMMNs

Based on theoretical and empirical insights from various studies, design principles for high‐performance electrocatalysts have been developed. The performance of nanocatalysts is sensitive to their physical and chemical characteristics, which necessitates employing appropriate synthetic procedures with reliability and reproducibility. This section describes the synthetic approaches for various kinds of NMMNs, major unsolved issues in the synthesis, and recent progress. We have classified NMMNs into six categories to present the principal synthetic methods in a more organized manner: random alloys, single‐atom alloys (SAAs), high‐entropy alloys (HEAs), ordered‐intermetallics, core–shell structures, and other heterostructures (Figure 1). Representative synthetic methods for a specific structure are described in each section with exemplary cases.

### 2.1 Random Alloys

A random alloy refers to a solid solution comprising multiple metal components with a random distribution in the crystal structure. This section includes the synthetic methods prevalently used for random alloy nanoparticles, followed by the modulation of the size, composition, and morphology of these nanoparticles. The last topic of this section concerns strategies to prevent phase separation of alloys to obtain homogenous single phases.

#### 2.1.1 Common Synthetic Routes

This section discusses the representative routes to synthesize random alloy nanoparticles together with exemplary works. Although the boundaries of the methods are being blurred as they have been modified and combined with each other as per the requirement, this section may help understand the synthetic factors that mainly affect the physical and chemical properties of nanoparticles and how they can be carefully controlled.

**Figure 2:**
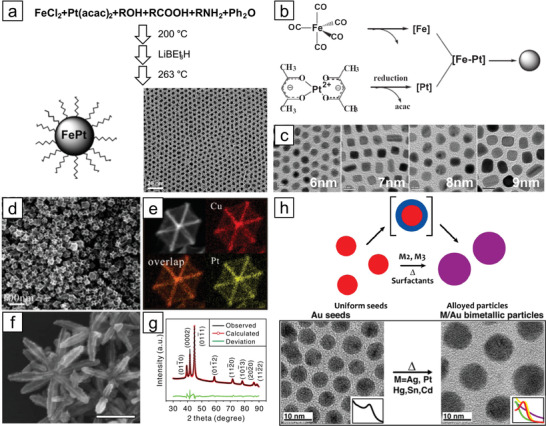
Representative synthetic methods for random alloy NMMNs. a) Coreduction of metal precursors using a reductant to achieve alloy nanoparticles: FeCl_2_ and Pt(acac)_2_ coreduced by LiBEt_3_H. Reproduced with permission.^[^ ^18^ ^]^ Copyright 2003, American Chemical Society. b) Schematic representation of thermal decomposition of Fe(CO)_5_ coupled with reduction of Pt(acac)_2_ for the synthesis of PtFe nanoparticles. Reproduced with permission.^[^ ^22^ ^]^ Copyright 2006, Wiley‐VCH. c) Transmission electron microscopy (TEM) images of as‐synthesized PtFe nanoparticles with size tuned from 6 to 9 nm. Reproduced with permission.^[^ ^23^ ^]^ Copyright 2004, American Chemical Society. d) High‐angle annular dark‐field scanning transmission electron microscopy (HAADF‐STEM), and e) energy dispersive X‐ray spectroscopy (EDS) mapping images of PtCu_3_ nanoparticles with excavated rhombic dodecahedral shape. Reproduced with permission.^[^ ^24^ ^]^ Copyright 2014, American Chemical Society. f) HAADF‐STEM image, and g) X‐ray diffraction (XRD) pattern of PtNi excavated nanomultipods with hexagonal close‐packed structure. Reproduced under the terms of the Creative Commons CC BY license.^[^ ^25^ ^]^ Copyright 2017, The Authors. Published by Springer Nature. h) Schematic diagram of seed‐mediated route to prepare random alloy nanoparticles and the corresponding TEM images. Reproduced with permission.^[^ ^30^ ^]^ Copyright 2015, American Chemical Society.

*Co‐reduction*: Simultaneous reduction of metal precursors is a straightforward route to prepare random alloy nanoparticles, especially when the alloyed metal elements have similar reduction potentials. In this process, supersaturation of monomers is induced by the introduction of an appropriate reducing agent, such as hydrazine, NaBH_4_, or polyol, into the precursor solution, followed by the nucleation and growth processes. For instance, **Figure** **2**a displays monodispersed PtFe nanoparticles obtained via coreduction of FeCl_2_ and Pt(acac)_2_ (acac = acetylacetonate) by introducing a reductant, LiBEt_3_H, with oleic acid and oleylamine as stabilizing agents.^[^ ^18^ ^]^ Another exemplary work is the synthesis of Pd‐based alloys,^[^ ^19^ ^]^ where a solution of mixed metal acetylacetonate precursors was injected into a preheated solution containing a reductant, such as borane morphine or borane *tert*‐butylamine, which reduces the precursors to PdCo and PdCu alloy nanoparticles.

*Thermal Decomposition*: In this approach, the synthesis of random alloy nanoparticles is triggered by the decomposition of organometallic compounds to monomers at high temperatures followed by nucleation of the supersaturated monomers. The hot‐injection and heat‐up processes fall under the category of thermal decomposition.^[^ ^20^ ^]^ The primary difference between these two processes is the way of achieving high supersaturation levels of monomers for a burst nucleation. In the hot‐injection method, the organometallic complexes are rapidly injected into an already heated reaction solution to induce burst nucleation. On the other hand, in the heat‐up method, precursors are mixed with all the other chemicals before heating up, and the supersaturation of monomers is achieved by continuous elevation of temperature. For this reason, hot‐injection method features flexibility for versatile syntheses, including the use of labile precursors, while heat‐up method features convenience by the absence of external operation during the reaction. As this technique requires high temperatures, the organic solvents of high boiling points are usually employed. The high temperature used in this method has merits in the selection of elements, enabling the use of metals such as Fe, Co, and Ni, which have relatively lower reduction potentials than precious metals. A representative case is the synthesis of PtFe nanoparticles introduced by Sun and co‐workers.^[^ ^21^, ^22^, ^23^ ^]^ The preparation of monodisperse PtFe nanoparticles was accomplished by the decomposition of Fe(CO)_5_ by injecting it into a solution containing a Pt precursor at high temperature and simultaneous reduction of the Pt precursor by diol (Figure 2b,c).

*Solvothermal Synthesis*: Solvothermal synthesis generally takes place at temperatures higher than the boiling point of the solvent and at pressures above atmospheric pressure to induce the reaction of the precursors. The reaction is usually performed in a Teflon‐lined autoclave, a closed vessel that can withstand high pressure and temperature. This method is useful when the synthesis of nanocrystalline materials requires better control over size and shape. For example, the synthesis of PtCu_3_ nanoparticles possessing an excavated rhombic dodecahedral shape could be accomplished via the reaction of acetylacetonate precursors in an autoclave in the presence of n‐butylamine and cetyltrimethylammonium chloride (CTAC) (Figure 2d,e).^[^ ^24^ ^]^ The authors suggested that the amine group in n‐butylamine critically contributes to the stabilization of the {110} facet of the nanoparticles, which is known to be the least stable among low‐index facets. Through the solvothermal technique, nanocrystals with metastable phases could also be prepared. The synthesis of Pt–Ni excavated nano‐multipods with metastable hexagonal close‐packed structures using formaldehyde and a Ni‐rich precursor is one of the examples (Figure 2f,g).^[^ ^25^ ^]^

*Incipient Wetness Impregnation*: Incipient wetness impregnation (IWI) is one of the simplest and most commonly employed methods for preparing metal nanoparticles anchored on support materials. A solution containing metal precursors is first mixed with support, and then the composite is completely dried to form supported nanoparticulates upon annealing. Despite the limited controllability of the size and composition of individual nanoparticles, the method has been used to prepare supported alloy nanoparticles owing to the ease and scalability. Recently, Abruña and co‐workers reported a generalized synthesis of RuM (M = Co, Ni, Fe) nanoparticles via wet impregnation of precursors into carbon support, followed by thermal annealing.^[^ ^26^ ^]^ Furthermore, the method can be extended to the synthesis of trimetallic nanoparticles by using precursors of three different metals.^[^ ^27^, ^28^ ^]^

*Seed‐Mediated Synthesis*: Seed‐mediated synthesis involves a two‐step process comprising the preparation of seed nanoparticles and incorporation of secondary (or more) metal species on seeds forming random alloy nanoparticles. The prerequisites to achieving homogeneous random mixing of metals are similar physicochemical properties of the constituent metals and sufficiently high temperatures. Seed‐mediated synthesis methods can be divided into two types: direct diffusion of secondary metals into seed particles and conversion of core–shell nanoparticles into random alloys. Sun and co‐workers successfully synthesized PdCu nanoparticles by controlling the diffusion of Cu into Pd nanoparticles at a high temperature (280 °C).^[^ ^29^ ^]^ Murray and co‐workers reported a generalized approach for thermal conversion of core–shell structures into random alloys starting from Au seeds (Figure 2h).^[^ ^30^ ^]^ Interestingly, the temperature required for the complete diffusion of each secondary metal (Ag, Pt, Hg, Sn, or Cd) varies from metal to metal. Similarly, Ag@Au^[^ ^31^ ^]^ and Au@Ag^[^ ^32^ ^]^ core@shell nanoparticles can be converted into randomly alloyed nanoparticles.

#### 2.1.2 Control over Size and Composition

The catalytic performance is largely affected by the size and composition of nanoparticles. These two parameters are sensitive to the reaction conditions, such as the concentration of precursors, stabilizing agent, solvent, reductant, and reaction temperature.

**Figure 3:**
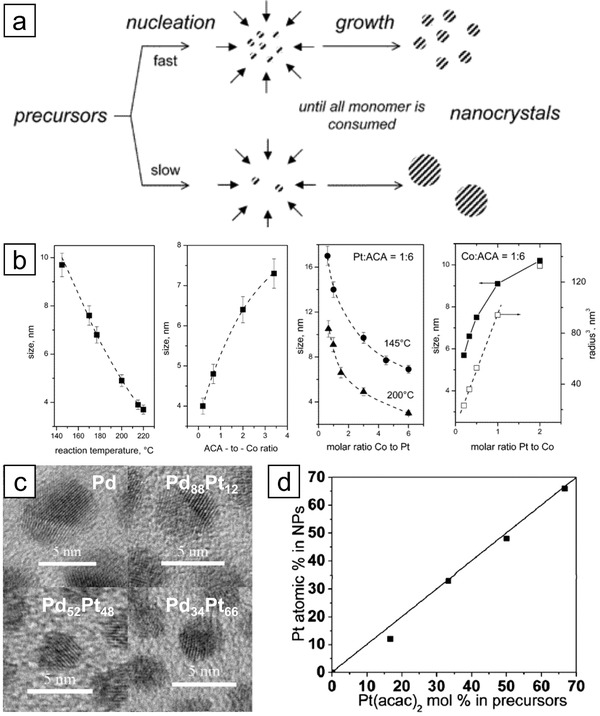
Control over the size and composition of NMMNs. a) Schematic representation of a relationship between nucleation rates and size of resultant nanoparticles in the absence of Ostwald ripening. b) Dependence of the size of Pt_3_Co on the reaction temperature, the ratio of surfactant to precursor, and the molar ratio between precursors. Reproduced with permission.^[^ ^34^ ^]^ Copyright 2003, American Chemical Society. c) TEM images of Pd and PdPt nanoparticles with controlled composition, and d) correlation between the proportion of Pt precursor and atomic percent of Pt in the alloy nanoparticles. Reproduced with permission.^[^ ^35^ ^]^ Copyright 2011, American Chemical Society.

The size of the nanoparticles is primarily determined in the nucleation and growth stages, especially by the number of nuclei and the total amount of precursor (**Figure** **3**a).^[^ ^33^, ^34^ ^]^ That is, the regulation of reaction rates gives rise to the variation in size. The effect of various control factors on the size of nanoparticles was extensively investigated in the synthesis of Pt_3_Co nanoparticles as a model system (Figure 3b).^[^ ^34^ ^]^ The alloying of Pt with Co can be achieved by injecting cobalt carbonyl into a preheated Pt precursor solution, and the high temperature induces the thermal decomposition of cobalt carbonyl, triggering the nucleation. The size of the nanoparticles was influenced by control factors such as injection temperature, the ratio of precursor to stabilizing agent, and the ratio of two precursors. As the injection temperature increased from 145 to 220 °C, the size decreased from 10 to 3 nm as more nuclei are produced. Similarly, the nucleation rate could be regulated by adjusting the amount of stabilizing agent as more stabilization decreases the supersaturation and suppresses the nucleation, resulting in the larger particles. Interestingly, the molar ratio between the precursors also affects the size. The smaller particle size obtained by a larger amount of Co is ascribed to the larger number of nuclei formed due to the increased nucleation rate. On the contrary, an increased concentration of Pt leads to the formation of larger particles as the precursor amount is increased. On the other hand, the composition of nanoparticles can be readily tuned by regulating the molar ratio of metal precursors. For example, Sun and co‐workers successfully synthesized composition‐controlled PdPt nanoparticles by varying the molar ratio of Pd and Pt precursors (Figure 3c,d).^[^ ^35^ ^]^ This strategy has been applied to different synthetic routes that control the composition of alloy nanoparticles.^[^ ^36^, ^37^ ^]^ Unfortunately, the size and composition are coupled to each other and are not independently tuned in most of the cases because of the intricate interactions among several control factors.

#### 2.1.3 Facet Control

The synthesis of multimetallic nanoparticles with well‐controlled morphology is important in many catalytic reactions since the differences in exposed facets affect the sorption properties of reactants, intermediates, and products, which change the overall catalytic performance.^[^ ^38^ ^]^ Typically, the evolution of a specific facet is determined in the growth stage, and the surface energy is critical for the resultant shape of the nanoparticles. If a facet possesses lower stability than other facets, the growth or coalescence over the facet takes place with fast kinetics for minimizing the overall surface energy of the nanoparticles, leading to diminution or even elimination of the facet.^[^ ^39^ ^]^ As a result, surfaces with lower surface energies tend to remain and to be exposed dominantly. The orientation of the facet can be guided by the chemicals involved in the reaction that have specified affinity toward certain facets, and the extent of facet dominance can also be controlled by the reaction temperature and time.

**Figure 4:**
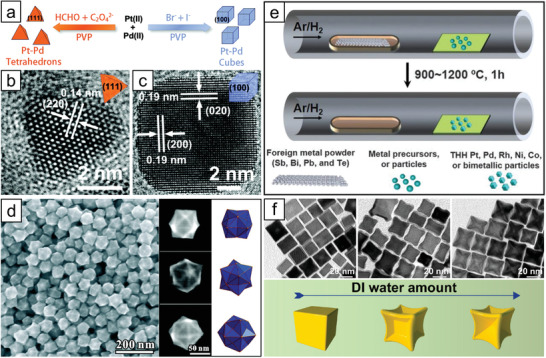
a) Schematic illustration of facet‐selective synthesis of PtPd nanoparticles, and TEM images of corresponding b) tetrahedral and c) cubic nanoparticles. Reproduced with permission.^[^ ^40^ ^]^ Copyright 2011, American Chemical Society. d) Electron microscopy images of HOH AuPd nanoparticles synthesized in the presence of OTAC and Cu^2+^ ions. Reproduced with permission.^[^ ^49^ ^]^ Copyright 2011, American Chemical Society. e) Scheme for inducing HIF on nanoparticles via alloying/dealloying route. Reproduced with permission.^[^ ^55^ ^]^ Copyright 2019, AAAS. f) Control over concavity of Pt_3_Co nanocubes by the amount of deionized water. Reproduced with permission.^[^ ^58^ ^]^ Copyright 2017, American Chemical Society.

Yan and co‐workers studied the facet‐selective synthesis of PtPd alloy nanoparticles and investigated the effect of chemical species.^[^ ^40^ ^]^ Although poly(vinylpyrrolidone) (PVP) acts as a surface‐stabilizing agent to prevent the aggregation of nanoparticles, it shows lacking facet‐directing ability. Instead, halide ions (for cubic nanoparticles) and a combination of HCHO with C_2_O_4_^2−^ (for tetrahedral nanoparticles) worked critically to determine the facet (**Figure** **4**a–c). Similarly, in another report, the presence of HCHO with C_2_O_4_^2−^ induced the formation of (111) facet‐dominant (icosahedral) nanoparticles, verifying the facet‐specificity of the chemicals.^[^ ^41^ ^]^ Although C_2_O_4_^2−^ commonly induces the exposure of (111) facet as in the case of PtPd tetrahedra, the evolution of multiply‐twinned icosahedral shape, instead of single‐crystalline tetrahedral shape, was observed. The deceleration in the reduction rate by the decreased amount of HCHO promoted the formation of multiply twinned metal nanocrystals (icosahedra). The role of halide ions in the shape control of PdPt alloy nanoparticles was further studied by Huang and co‐workers.^[^ ^42^ ^]^ In this study, NaCl and NaI were added to the reaction solution, and the addition resulted in nanoparticles terminated with (111) and (100), respectively. Interestingly, when halide ions (Cl^−^, Br^−^, I^−^) were mixed together, the iodide ion produced cubic nanoparticles regardless of the other halide ions. Another shape‐directing agent investigated systematically is CO, which has been commonly used for Pt‐based nanoparticles with controlled shapes.^[^ ^43^, ^44^ ^]^ In the synthesis of Pt_3_Ni cubes and octahedrons, the shape of the nanoparticles became relatively irregular without CO, implying the significance of CO than oleic acid and oleylamine for shape control.^[^ ^45^ ^]^ In another study conducted by Peng and co‐workers, the amount of Ni precursor was adjusted to obtain octahedral‐shaped PtNi nanoparticles by applying the preferential adsorption of CO for (100) on Pt and (111) on Ni.^[^ ^46^ ^]^

Among the shape‐controlled nanocatalysts, multimetallic nanoparticles with high‐index facets (HIFs) have attracted much attention owing to their low‐coordinated surface atoms that exhibit enhanced activity for several electrocatalysis.^[^ ^47^, ^48^ ^]^ However, the instability of the facets makes it difficult for the facets to survive under reaction conditions. While extensive progress has been made in the fabrication and systematic studies on monometallic systems with HIF, there are very few studies for multimetallic systems, owing to the higher complexity compared to monometallic systems.

The most prevalent strategies for the fabrication of HIF‐bound NMMNs are introducing facet‐directing agents and controlling the reaction kinetics of the precursors. One pioneering work is the synthesis of hexoctahedral (HOH) AuPd nanoparticles (Figure 4d).^[^ ^49^ ^]^ The synthesis was aided by the presence of octadecyltrimethylammonium chloride (OTAC) and Cu^2+^ ions, which facilitated the formation of the HOH shape and guaranteed the homogeneous phase by underpotential deposition, respectively. Further, OTAC is used as a capping agent in the synthesis of rhombic dodecahedral and trisoctahedral AuPd nanoparticles.^[^ ^50^ ^]^ During the synthesis of PtNi, Sun and co‐workers identified the role of glycine in the HIF structure in the presence of PVP, and obtained the morphological evolution from concave nanocubes to nanocubes to hexoctahedra with the increase in glycine content.^[^ ^51^ ^]^ They attributed the formation of HIFs to the lowered reduction rate of metal ions through strong coordination with glycine, thereby changing the nucleation and growth rates. Moreover, the combination of glycine and PVP has been utilized to synthesize other Pt‐based bimetallic and trimetallic alloy nanoparticles with controlled HIF.^[^ ^52^, ^53^ ^]^

Mirkin and co‐workers discovered that the exposed facets of gold nanoparticles could be diversified from low‐index to high index by adjusting the added amount of shape‐directing silver species in solution‐phase synthesis.^[^ ^54^ ^]^ Recently, they extended the use of foreign shape‐directing elements for the evolution of HIF on mono‐ and bimetallic nanoparticles in a general and scalable manner via solid‐state synthesis.^[^ ^55^, ^56^ ^]^ The whole procedure consists of two steps: colloidal synthesis of noble‐metal‐based nanoparticles with irregular shapes followed by thermal treatment in the presence of foreign metals, such as Sb, Bi, Pb, and Te, to induce shape directing via alloying/dealloying (Figure 4e).

It has been reported that selective etching is an effective strategy.^[^ ^57^ ^]^ Shen and co‐workers reported the construction of deeply excavated Pt_3_Co nanocubes via oxidative etching by Cl^−^/O_2_ pairs during a solvothermal synthesis.^[^ ^58^ ^]^ The introduction of water into the reactor during the synthesis is critical for excavating surfaces because the presence of water breaks the kinetic equilibrium of surface capping CTAC and provides more O_2_, enhancing the corrosion rate (Figure 4f).

#### 2.1.4 Random Alloys with Hollow Structures

One special morphology that has garnered much attention of researchers is the hollow (or open) structure due to its intrinsically large surface area, which makes it easy to maximize the atomic efficiency for catalysis. The fabrication of hollow nanoparticles is usually accomplished by template‐mediated routes. The template can be either presynthesized or in situ formed during the synthesis, and later removed by etching, replacement, or migration by the Kirkendall effect, leaving a cavity inside a nanoparticle.

**Figure 5:**
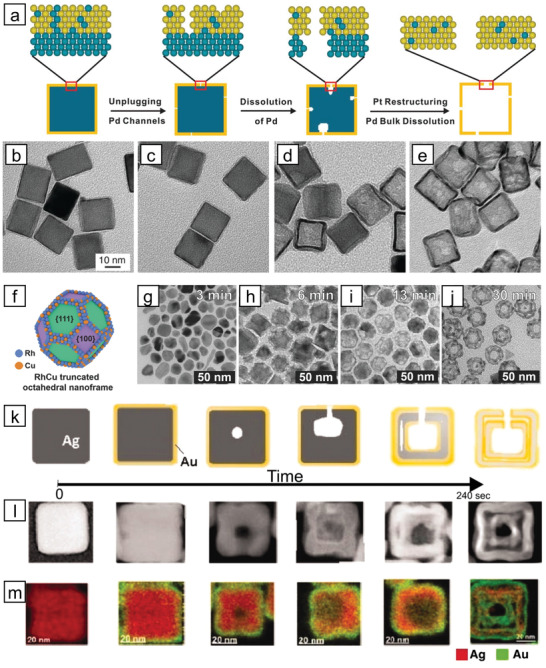
a) Detailed mechanism of void formation inside the Pd@Pt core–shell nanocubes by selective etching. TEM images of Pd@Pt nanocubes etched for b) 0 min, c) 10 min, d) 30 min, and e) 180 min. Reproduced with permission.^[^ ^59^ ^]^ Copyright 2015, AAAS. f) Ideal model structure of a RuCu nanoframe and the temporal TEM images obtained at g) 3 min, h) 6 min, i) 13 min, and j) 30 min of reaction. Reproduced under the terms of the Creative Commons CC BY license.^[^ ^64^ ^]^ Copyright 2015, The Authors. Published by Wiley‐VCH. k) Structural evolution of AuAg dual‐walled nanoboxes over reaction time, and corresponding l) HAADF‐STEM, and m) EDS mapping images. Reproduced with permission.^[^ ^68^ ^]^ Copyright 2011, AAAS.

Etching is a popular method for removing templates to create voids.^[^ ^57^ ^]^ Xia and co‐workers utilized a chemical etching route to fabricate PtPd nanocages with cubic and octahedral shapes (**Figure** **5**a–e).^[^ ^59^ ^]^ An aqueous solution containing FeCl_3_ and HCl was used for the selective etching of the Pd core in Pd@Pt nanoparticles. The method was further applied to the synthesis of IrPd nanocages by etching Pd@Ir nanoparticles.^[^ ^60^ ^]^ On the other hand, composition‐segregated nanoparticles were employed as precursors instead of core@shell nanoparticles. For example, a polyhedral PtNi nanoframe was produced by etching the Ni‐rich phase with acetic acid to form nanoparticles with composition segregation.^[^ ^61^ ^]^ Similarly, PtCo nanoframes with rhombic dodecahedral morphology were obtained by chemical etching of Co in phase‐segregated PtCo nanoparticles using nitric acid.^[^ ^62^ ^]^

The galvanic replacement reaction refers to the oxidation of less noble metal atoms coupled with the reduction of more noble metal ions. The process is thermodynamically driven based on the difference between the reduction potentials of the metals involved. Because less noble metals are sacrificed during the process, the galvanic replacement has been exploited to achieve nanoparticles with hollow shapes. For example, in situ formed Co nanoparticles were utilized as sacrificial templates for galvanic replacement with Pt ions.^[^ ^63^ ^]^ In the synthesis, Co remains and participates as a composing metal after being consumed as a template. It is suggested that, in the presence of PVP, which is known to trap metal cations, the excessive reducing environment by a remnant reductant allowed the formation of PtCo alloy through the coreduction of Pt ions and the leached Co ions. The shapes of the hollow nanoparticles were also readily tuned by a slight modification of the reaction conditions in the reactor. Lee and co‐workers synthesized a series of RhCu nanoframes with various morphologies via galvanic replacement of the Rh precursor onto Cu nanoparticles (Figure 5f–j).^[^ ^64^ ^]^ Control of reductants or temperature during galvanic replacement affects the shapes of the templates and the resulting nanoframes. Xia and co‐workers demonstrated that the thickness of a hollow nanostructure could be precisely controlled via regeneration of a template, followed by galvanic replacement, using Ag–Au cubic nanocages as a model case.^[^ ^65^ ^]^ First, Ag–Au nanocages were prepared by galvanic replacement over sacrificial Ag nanocubes. The hollow structure was refilled with Ag core by l‐ascorbic acid as a reductant, forming an Ag@Ag–Au core–shell structure, and then further galvanic replacement was conducted using these core–shell particles as a new template. After repeated reactions, the thickness of the nanoparticles could be regulated, and this technique was successfully applied to other combinations of metals and trimetallic systems.

The Kirkendall effect, driven by the difference in the diffusion rates of metals at the interface, can be utilized to fabricate hollow NMMNs, when the diffusion rate of core metal is higher than that of shell component. Yang and co‐workers investigated the structural evolution of Cu@Pt core–shell nanoparticles in toluene for three weeks under ambient conditions with the controlled kinetics.^[^ ^66^ ^]^ Over time, the Cu atoms diffused toward the shell at a higher rate than the inward diffusion of Pt, leaving voids inside the nanoparticles and converting them into PtCu_3_ nanoframes. The Kirkendall effect can be accelerated by increasing the diffusion rate of core metals at a sufficiently high temperature. For example, hollow cavities in the PtFe nanoparticles were constructed via the thermal treatment (800 °C for 2 h) of Pt nanoparticles covered by an Fe^3+^‐chelating polydopamine layer.^[^ ^67^ ^]^ The higher outward diffusion of Pt than the inward diffusion of Fe resulted in hollow PtFe nanoparticles. The simultaneous application of galvanic replacement and the Kirkendall effect could produce interesting hollow structures. The synthesis of concentric double‐walled AuAg nanoboxes was achieved by sequential galvanic replacement and the Kirkendall effect.^[^ ^68^ ^]^ The voids inside the Ag templates were produced by the leaching of Ag^+^ ions by galvanic replacement with Au ions at the outer surface. Once the voids formed, galvanic replacement between Au and Ag also occurred at the inner voids, constructing two separate Au layers on the inside and outside surfaces of the hollow Ag frame, which finally evolved into double‐walled nanoboxes by the Kirkendall effect (Figure 5k–m).

#### 2.1.5 Prevention of Phase Segregation

Generally, precursors with similar reduction rates easily form homogenous alloy nanoparticles. For example, PdAg alloy nanoparticles were readily formed because of the similar reduction potential of Ag with Pd (+0.8 V for Ag^+^/Ag and +0.9 V for Pd^2+^/Pd).^[^ ^69^ ^]^ Unfortunately, not all combinations of metals are eligible because a huge difference in the reduction rate leads to a sequential reduction of metals, producing phase‐segregated products. This problem can be tackled in two ways: introducing additional chemicals or using bimetallic complexes.

**Figure 6:**
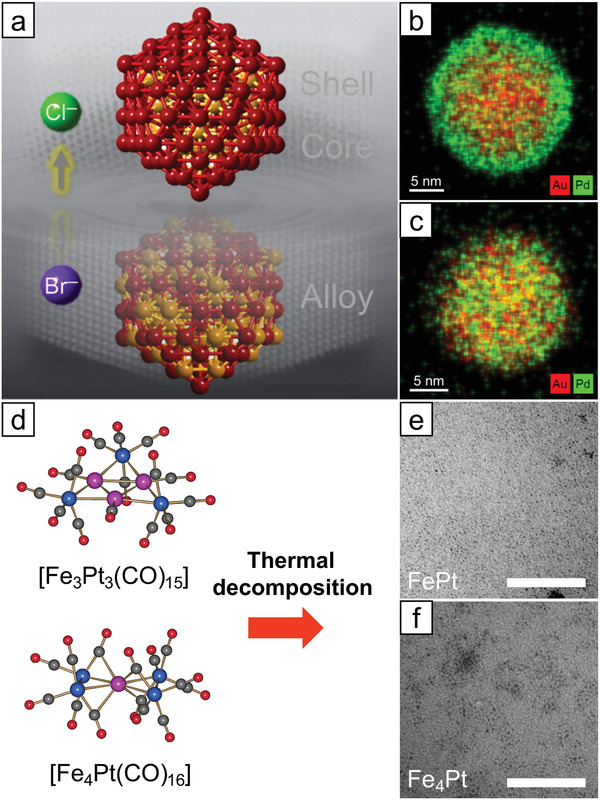
a) Schematic diagram for describing an aspect of nanoparticle formation in the presence of halide species with different ratios. EDS mapping images of b) AuPd core–shell nanoparticles in Cl−rich environment and c) random alloy in Br−rich one. Reproduced with permission.^[^ ^71^ ^]^ Copyright 2016, American Chemical Society. d) Structures of dual metal precursors with controlled metal ratio, and e,f) TEM images of corresponding nanoparticles produced by thermal decomposition (scale bar: 100 nm). Reproduced with permission.^[^ ^74^ ^]^ Copyright 2009, American Chemical Society.

As the precursor reduction rates are closely related to the reduction potential and stability of metal ions, they have been effectively regulated by the introduction of additional chemicals. Xia and co‐workers showed that the presence of KBr significantly affected the compositional homogeneity of Pd–Pt nanoparticles after coreduction.^[^ ^70^ ^]^ Cubic‐shaped homogeneous PdPt random alloys were produced with a KBr additive, while octahedral‐shaped Pd@Pt nanoparticles were formed in the absence of KBr. In the presence of KBr, PtCl_4_^2−^, and PdCl_4_^2−^ transformed into PtBr_4_^2−^ and PdBr_4_^2−^ because of the stronger binding strengths of Br^−^ than Cl^−^ toward the metal ions. The ligand exchange narrowed the gap of the reduction rates between the Pt and Pd precursors by slowing them down. Kuo and co‐workers have demonstrated that the reduction of HAuCl_4_ and H_2_PdCl_4_ by sodium citrate can be controlled by varying the amounts of CTAC and cetyltrimethylammonium bromide (CTAB) additives.^[^ ^71^ ^]^ By changing the ratio of Br^−^ to Cl^−^ (in CTAB and CTAC), core–shell nanoparticles were produced in the Cl^−^‐rich case, while random‐alloy nanoparticles were prepared in the Br^−^‐rich case (**Figure** **6**a–c). The above results indicate that additives can play pivotal roles in the synthesis of random alloys by controlling the reduction rates of the precursors. Moreover, the reduction rates of metal precursors can also be affected by their concentrations. Sun and co‐workers reported that similar rates for the nucleation and growth of Ag and Au precursors could be achieved by using a much larger amount of silver precursor (20 times) than Au, which compensated for the much higher reduction potential of Au (1.5 V for Au^3+^/Au).^[^ ^72^ ^]^

Another strategy for overcoming this difference is the use of a bimetallic precursor instead of a mixture of monometallic precursors. Lukehart and co‐workers first reported the formation of PtFe nanoparticles using a bimetallic precursor.^[^ ^73^ ^]^ The Fe_3_Pt_3_(CO)_15_ complex, prepared by reacting Fe(CO)_5_ with Pt(C_7_H_10_)_3_, underwent ultrasonication to produce precipitates of PtFe nanoparticles with nearly 1:1 composition. Thanh and co‐workers extended the synthesis of PtFe and PtFe_4_ nanoparticles by the thermal decomposition of [Fe_3_Pt_3_(CO)_15_]^2−^ and [Fe_4_Pt(CO)_16_]^2−^, respectively (Figure 6d–f).^[^ ^74^ ^]^

### 2.2 Single‐Atom Alloys

SAA is a type of single‐site catalyst in which a trace amount of catalytically active metal is atomically dispersed on the surface of the host metal. Owing to the low coordination, isolation, and well‐defined nature of active sites, SAAs have shown excellent selectivity as well as outstanding atomic efficiency, attracting attention from researchers as good model catalysts for various kinds of reactions.^[^ ^75^ ^]^ In addition to their role as model systems, SAAs in the form of nanoparticles have recently been utilized in the fields of practical applications, such as electrochemical water splitting and fuel cell catalysis.^[^ ^76^ ^]^ The synthesis of SAA nanoparticles with various routes has been investigated over the past several years to fully exploit the above‐mentioned advantages of SAA nanoparticles. In fact, the representative synthetic methods for SAA nanoparticles do not vary much from those for random alloys and core–shell nanoparticles. For the synthesis of SAA nanoparticles, the amount of the dopant species should be much lower than that of the host so that the dopant atoms are completely isolated as single atoms on the host. On the other hand, the synthesis of random alloys or core–shell nanoparticles requires sufficiently higher amount of dopants than that of SAA nanoparticles.

**Figure 7:**
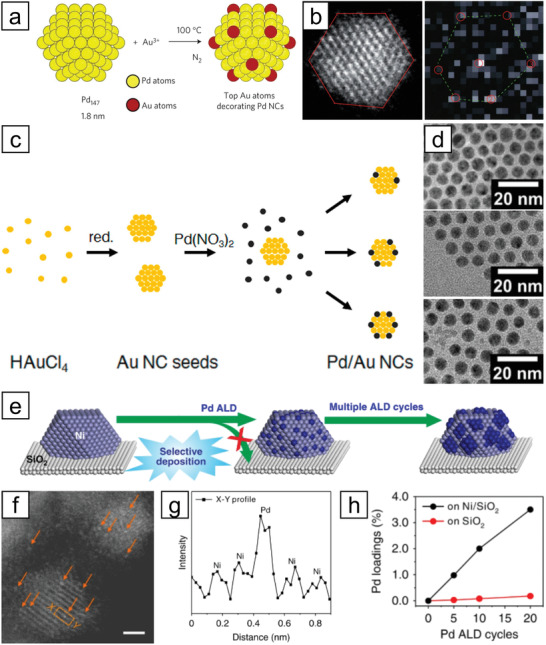
a) Schematic illustration of the formation of Au_1_/Pd SAA nanoparticles via galvanic replacement of Au^3+^ ions and surface Pd atoms. b) HAADF‐STEM and electron energy loss spectroscopy (EELS) images of Au_1_/Pd. Bright dots marked by red circles in the EELS image indicate the presence of Au single atoms. Reproduced with permission.^[^ ^77^ ^]^ Copyright 2011, Nature Publishing Group. c) Schematic illustration of sequential reduction of Au and Pd for the fabrication of Pd_1_/Au SAA nanoparticles with varied Pd loading, and d) the TEM images of corresponding resultants. Reproduced with permission.^[^ ^92^ ^]^ Copyright 2018, American Chemical Society. e) Schematic illustration of selective deposition of Pd atoms on a Ni nanoparticle through ALD technique. f) HAADF‐STEM image for Pd_1_/Ni SAA nanoparticles (Scale bar: 2 nm). g) The profile of line *X*–*Y* shows the single atomic feature. h) Pd loading on the Pd/Ni samples as a function of ALD cycle number. Reproduced under the terms of the Creative Commons CC BY license.^[^ ^104^ ^]^ Copyright 2019, The Authors, published by Springer Nature.

Toshima and co‐workers reported the first synthesis of SAA nanoparticles using galvanic replacement.^[^ ^77^ ^]^ They prepared isolated Au sites on Pd clusters by adding an aqueous solution of HAuCl_4_⋅4H_2_O into dispersed Pd clusters. As the surface free energies of Pd atoms on Pd clusters differed according to their positions, Au preferentially displaced the Pd atoms at specific sites (**Figure** **7**a,b). Copper is one of the most intensively used hosts in the synthesis of SAA nanoparticles via galvanic replacement. Flytzani‐Stephanopoulos and co‐workers published a series of papers on the preparation of Pd_1_/Cu^[^ ^78^ ^]^ and Pt_1_/Cu^[^ ^79^, ^80^, ^81^, ^82^ ^]^ nanoparticles by galvanic replacement of Cu. Wei and co‐workers also used Cu nanoparticles as a host for synthesizing Pt_1_Cu SAA nanoparticles.^[^ ^83^ ^]^ Besides spherical nanoparticles, Cu nanosheets and nanocubes were also used as host materials for galvanic replacement with Pd.^[^ ^84^ ^]^ With the different morphologies of the hosts, the catalytic properties of Pd_1_Cu were significantly adjusted according to the different exposed facets. Wasserscheid and co‐workers used Ga nanoparticles as host materials and successfully synthesized Pd^[^ ^85^ ^]^ and Rh^[^ ^86^ ^]^ single‐atom alloys on a Ga host using (NH_4_)_2_[PdCl_4_] and RhCl_3_·H_2_O as precursors, respectively. Similarly, the synthesis of SAA using Ni as a host was also achieved by Zeng and co‐workers.^[^ ^87^ ^]^ It is important to note that the precursor concentration of a doped metal should be carefully adjusted. Sykes and co‐workers^[^ ^79^ ^]^ controlled the concentration of Pt in the solution for galvanic replacement with Cu nanoparticles. From the EXAFS analysis, the formation of isolated Pt single‐atomic sites was confirmed by the absence of Pt–Pt bond for the sample prepared with low Pt concentration, while one with higher Pt concentration showed the presence of Pt–Pt bond.

Unfortunately, the galvanic replacement method can be used only when the dopant metal is more noble than the host nanoparticles. On the other hand, a sequential reduction can be a suitable alternative if a noble metal host is to be used. In sequential reduction, one metal species is reduced first to form seed nanoparticles, followed by the reduction of a secondary metal onto the surface of the seeds. To date, most reports have used Au nanoparticles as the seed material, with Pd as a dopant. Various combinations of precursors and reductants have been employed for sequential reduction: NaBH_4,_^[^ ^88^ ^]^ ethylene glycol,^[^ ^89^, ^90^ ^]^ ascorbic acid,^[^ ^91^ ^]^ and oleylamine^[^ ^92^ ^]^ for Pd(NO_3_)_2_, formic acid for H_2_PdCl_4_,^[^ ^93^ ^]^ and H_2_ bubbling for PdCl_2_^[^ ^94^ ^]^ (Figure 7c,d).

All the aforementioned methods require a multistep procedure in the solution phase, with complexity, cost, and time. Co‐reduction provides a facile route for obtaining SAA nanoparticles. A representative example was reported by Shishido and co‐workers.^[^ ^95^ ^]^ They synthesized Pd–Au alloy nanoparticles through coreduction method, and obtained Au_1_/Pd SAA, PdAu random alloy, Pd_1_/Au SAA nanoparticles by simply adjusting the ratio of Pd precursor to Au precursor under the same reaction conditions. The synthesis of several SAA nanoparticles such as Pd_1_/Ag,^[^ ^96^ ^]^ Cu_1_/Pd,^[^ ^97^ ^]^ Pd_1_/Au, and Rh_1_/Au,^[^ ^98^ ^]^ has been achieved by simply adjusting the molar ratio of precursors with suitable reductants.

As in the case of random alloys, incipient wetness impregnation also provides a simple way to synthesize SAA nanoparticles by controlling the ratio of impregnated metal precursors. Zhang and co‐workers reported a series of Pd‐based SAA nanoparticles with different Pd/Ag ratios on silica support by adjusting the molar ratio of Pd/Ag precursors from 0.005 to 0.025.^[^ ^99^ ^]^ Additionally, they reported the synthesis and catalytic application of Pd_1_/Cu nanoparticles on SiO_2_.^[^ ^100^ ^]^ They changed the ratio of Pd to Cu with fixed Cu loading or fixed Pd loading. In the same manner, *γ*‐Al_2_O_3_ was also used as a support material.^[^ ^101^, ^102^, ^103^ ^]^ Although IWI is a facile technique to prepare SAA nanoparticles, it should be noted that the resultant products usually have inhomogeneity in sizes and compositions from particles to particles, which can be a potential problem in particular cases where high uniformity is required.

Lastly, the atomic layer deposition (ALD) technique can also be used to prepare SAA nanoparticles. Lu and co‐workers reported the synthesis of Pd_1_/Ni nanoparticles on SiO_2_ support using ALD.^[^ ^104^ ^]^ With an increase in the number of ALD cycles, the loading of Pd in the nanoparticles increased, eventually losing the single atomic feature (Figure 7e–h). Although the metal atoms deposited by ALD cycles can form isolated atomic sites due to the steric hindrance by bulky ligands, aggregation of metal atoms often occurs after multiple ALD cycles.^[^ ^105^ ^]^ To prevent the aggregation of the deposited metal species, the Pd ALD was performed on Ni/SiO_2_ at a relatively low temperature (150 °C). Sun and co‐workers studied the formation of Pt single‐atomic sites on Pd nanoparticles using the ALD technique.^[^ ^106^ ^]^ Interestingly, isolated Pt atoms were embedded on octahedral Pd nanoparticles. At the same time, a few atomic layers of Pt were formed on cubic Pd nanoparticles under the same operating conditions, mainly due to the different surface energies of Pt (111) and Pt (100).

### 2.3 High Entropy Alloys

HEAs usually refer to alloys of multiple (five or more) elements with maximized entropy from nearly equal contents.^[^ ^107^ ^]^ Recently, HEAs have come into the spotlight, and the synthesis of HEAs in the nanometer size region has become important for maximizing the utilization of surface atoms in catalysis with various kinds of atomic ensembles.^[^ ^108^, ^109^, ^110^ ^]^

Unfortunately, traditional wet‐chemical routes used to synthesize bi‐ and trimetallic nanoparticles tend to produce phase‐segregated products of polyelemental systems,^[^ ^111^ ^]^ because of the differences in the reactivity of multiple metal precursors and immiscibility of metal elements. Therefore, ingenious synthetic methods featuring rapid heating/cooling have been developed to prepare polyelemental solid solutions with a reliably homogeneous phase. The rapid heating ensures simultaneous reduction of metal precursors with different chemical potentials, while rapid cooling also plays a pivotal role in preventing the phase separation among immiscible elements, thereby preventing a compositional inhomogeneity in the prepared HEA nanoparticles.^[^ ^112^ ^]^

**Figure 8:**
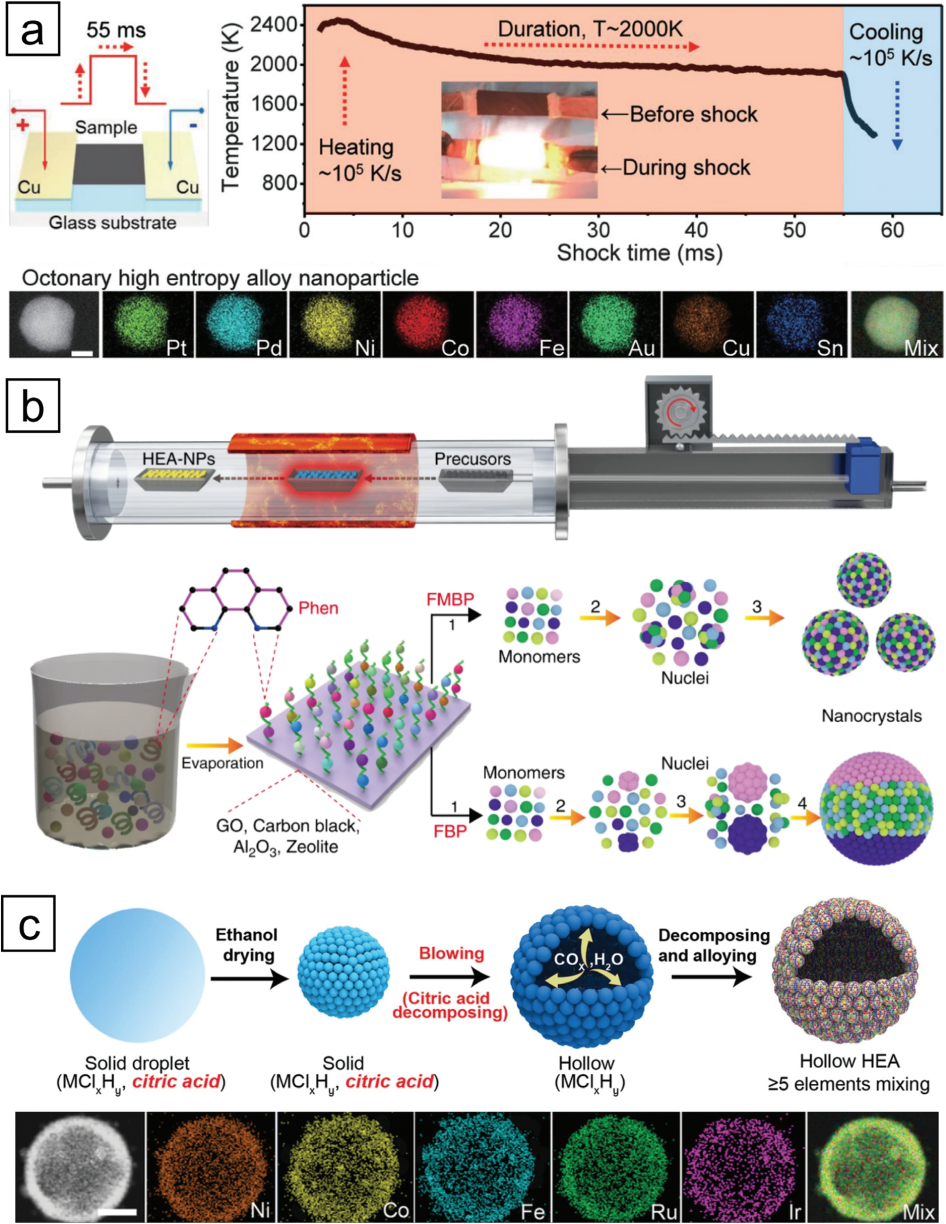
Representative synthetic approaches for HEA NMMNs. a) Schematic diagram of CTS method (top) and EDS mapping images of a representative HEA nanoparticle comprising eight elements (bottom, scale bar: 10 nm). Reproduced with permission.^[^ ^112^ ^]^ Copyright 2018, AAAS. b) Schematic illustration of fast‐moving bed approach, and comparison with the case using fixed‐bed. Reproduced under the terms of the Creative Commons CC BY license.^[^ ^119^ ^]^ Copyright 2020. Published by Springer Nature. c) Schematic illustration of the fabrication of hollow HEA nanoparticles via aerosol‐mediated synthesis (top) and the EDS mapping images of a representative nanoparticle (bottom, scale bar: 100 nm). Reproduced with permission.^[^ ^121^ ^]^ Copyright 2020, Wiley‐VCH.

Carbothermal shock (CTS) synthesis was shown to be an effective technique for synthesizing HEA nanoparticles, which was proposed by Hu and co‐workers (**Figure** **8**a).^[^ ^112^ ^]^ First, carbon nanofiber was immersed in a precursor solution for loading metal species. Then, a thermal shock (≈2000 K) was applied to the precursor‐loaded carbon nanofiber using Joule heating by an electrical pulse. They reported the successful synthesis of well‐dispersed nanoparticles comprising up to eight elements using this method. In the control experiments, prolonged duration at high temperatures produced larger particles, while a slower cooling rate led to phase segregation. A subsequent study compared the products synthesized using the CTS method with those from the IWI method.^[^ ^113^ ^]^ Although the morphology and composition of the two cases were not very different, the former showed superior stability to the latter during catalysis, while HEAs with a larger number of components further enhanced the stability. The reliability and generalizability of the technique have facilitated the synthesis of other HEA nanoparticles comprising various combinations of elements.^[^ ^114^, ^115^, ^116^, ^117^, ^118^ ^]^

Lu and co‐workers suggested that pyrolysis using a fast‐moving bed is a reliable synthetic method for synthesizing HEA nanoparticles on granular supports (Figure 8b).^[^ ^119^ ^]^ In this method, a boat containing precursor‐loaded support was moved into the center of the furnace preheated to 923 K with a propulsion speed of ≈20 cm∙s^−1^. When the fast‐moving bed passed through the heating zone, the temperature of the boat rapidly increased within only 5 s, inducing simultaneous pyrolysis of the precursors and formation of denary HEA nanoparticles. Contrary to the case of the fast‐moving bed, phase‐separated nanoparticles were obtained when using a fixed or slow‐moving bed because the metal precursors were reduced sequentially as the temperature increased. It is worth noting that this technique is generally adopted for various supports, such as carbon black, graphene oxide (GO), zeolite, and alumina.

The aerosol droplet‐mediated technique is a continuous process for the fabrication of HEA nanoparticles with high scalability. In this process, a solvent containing the precursors was nebulized and carried into a heating zone using a carrier gas. While passing through the heating zone, HEA nanoparticles were formed quickly, followed by rapid quenching after their removal from the zone. Zachariah and co‐workers first reported this method with excellent compositional homogeneity in a single nanoparticle.^[^ ^120^ ^]^ They were able to form alloys of several combinations of immiscible metals in a particle. Hu and co‐workers successfully synthesized hollow HEA nanoparticles by introducing a gas‐blowing agent (Figure 8c).^[^ ^121^ ^]^ Citric acid dissolved in ethanol rapidly decomposes to CO_2_ and H_2_O when the droplet passes through a heating zone, forming hollow cavities inside the nanoparticles. The aforementioned approaches collectively show that rapid heating and cooling induce simultaneous reactions of metal precursors, which otherwise would react at different rates.

Compared to the methods using rapid heating and cooling, the wet‐chemical route is disadvantageous because a gradual change in temperature induces a sequential reaction of precursors, which results in the inhomogeneous elemental distribution in each particle. Nevertheless, wet‐chemical approaches for the synthesis of HEA nanoparticles have been steadily investigated. Iversen and co‐workers prepared binary to quinary nanoparticles with homogeneous phases via a solvothermal method.^[^ ^122^ ^]^ They observed a single homogeneous phase with a face‐centered cubic (fcc) structure (which is unusual for Ru‐based alloys) by using metal acetylacetonate precursors. Additionally, as the reaction temperature exceeded a certain threshold, products with hcp structures were produced. An ultrasonication‐assisted method was also applied to synthesize PtAuPdRhRu HEA nanoparticles with diameters less than 3 nm.^[^ ^123^ ^]^ Under intense ultrasonication, acoustic cavitation conditions are generated with the explosion of bubbles transiently, creating extremely high pressures (≈2000 atm) and high temperatures (5000 °C) in a local region. With the massive amount of energy, the precursors were coreduced, forming HEA nanoparticles. Skrabalak and co‐workers demonstrated the synthesis of core–shell mediated HEA nanoparticles.^[^ ^124^ ^]^ They used PdCu@PtNiCo nanoparticles as a precursor for HEAs. The core–shell nanoparticles were deposited on carbon support to prevent coalescence and then annealed at high temperatures to promote the diffusion and intermixing of metal elements.

As in the case of monometallic and other heterostructured nanoparticles, it can be inferred that the size and exposed facets influence the catalytic performance of HEA nanoparticles. However, the control over the morphology of HEA nanoparticles has not been systematically studied, and the above‐mentioned routes still have intrinsic limitations in morphological control, because most of the current methods apply extremely high temperature, which makes it difficult to control the size and shape of HEAs. Therefore, a more advanced synthetic method is required for a better morphology control.

### 2.4 Ordered‐Intermetallics

Alloys with a regular atomic arrangement between constituent metals of more than two different types of elements are termed intermetallic alloys.^[^ ^125^ ^]^ Atomically ordered structures, such as L1_0_, L1_1_, L1_2_, and B2 phases, feature highly effective electronic (d–d orbital) interactions between different metal elements and stabilized metal elements (especially 3d transition metals) that are vulnerable to oxidation or leaching. Over the last two decades, tremendous efforts have been devoted to prepare intermetallic nanoparticles that are highly ordered and precisely tuned in composition and morphology.^[^ ^126^, ^127^ ^]^

Herein, we describe the developments in synthesizing intermetallic alloy nanoparticles that provide significant insights into a realization of desirable intermetallic nanomaterials. Our focus is not on listing possible synthetic methods but on describing key advancements in precisely controlling the structural properties of intermetallic nanoparticles. We begin our discussion on Pt intermetallics and then proceed to Pd and Au intermetallics.

#### 2.4.1 Pt Intermetallics

**Figure 9:**
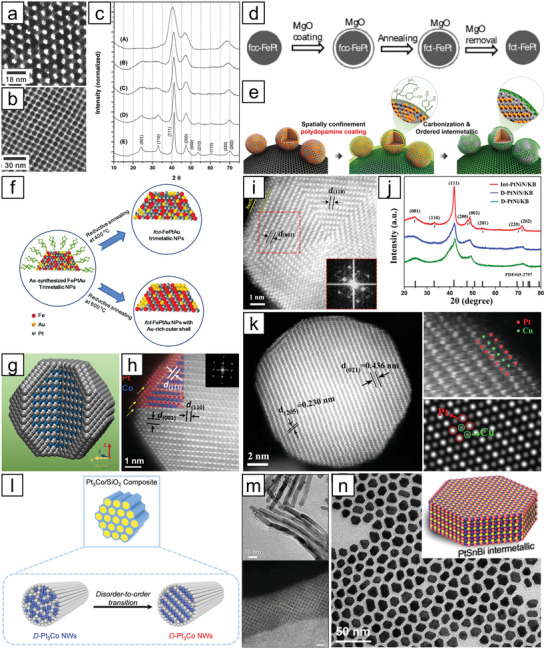
a,b) TEM images of assemblies of monodispersed 6 nm PtFe nanoparticles and c) XRD patterns of 4 nm PtFe nanoparticles annealed to form L1_0_‐PtFe phase. Reproduced with permission.^[^ ^21^ ^]^ Copyright 2000, AAAS. Schematic illustration of d) MgO‐coating approach. Reproduced with permission.^[^ ^132^ ^]^ Copyright 2009, Wiley‐VCH. e) Schematic illustration of N‐doped carbon coating approach. Reproduced with permission.^[^ ^134^ ^]^ Copyright 2015, American Chemical Society. f) Schematic illustration of structural evolution of PtFeAu upon annealing. Reproduced with permission.^[^ ^137^ ^]^ Copyright 2012, American Chemical Society. g) Schematic and h) STEM image of an L1_0_‐PtCo nanoparticle. Reproduced with permission.^[^ ^144^ ^]^ Copyright 2019, Elsevier. i) STEM image and j) XRD pattern of intermetallic PtNiN/KB. Reproduced with permission.^[^ ^150^ ^]^ Copyright 2020, American Chemical Society. k) HAADF‐STEM image (left), enlarged HAADF‐STEM image (right top) and simulated HAADF‐STEM image (right bottom) of intermetallic PtCuN nanoparticle. Reproduced with permission.^[^ ^153^ ^]^ Copyright 2021, American Chemical Society. l) Schematic illustration of template‐mediated synthesis of L1_2_‐Pt_3_Co nanowires and their m) TEM (top) and HAADF‐STEM (bottom) images. Reproduced with permission.^[^ ^165^ ^]^ Copyright 2019, American Chemical Society. n) TEM image of PtSnBi nanoplates and schematic illustration of its atomic arrangement (inset). Reproduced with permission.^[^ ^169^ ^]^ Copyright 2019, Wiley‐VCH.

Among all noble metal‐based intermetallic alloy nanoparticles, Pt–Fe intermetallics are most common and well approved. Sun and co‐workers first reported the precise synthesis of spherical PtFe nanoparticles and annealing of their assembly that has had a profound impact on the emergence of several succeeding works on intermetallic nanoparticles with desired properties.^[^ ^21^ ^]^ The spherical PtFe nanoparticles were self‐assembled into hexagonal close‐packed or cubic packed structures on a substrate (**Figure** **9**a,b), followed by thermal annealing in an inert gas that allowed the rearrangement of the constituent metal atoms. Eventually, it led to atomically ordered PtFe nanoparticles with the L1_0_ phase (face‐centered tetragonal) (Figure 9c). Since then, the postannealing process on presynthesized random alloy nanoparticles has been the main strategy to obtain ordered Pt‐alloy nanoparticles, while the size of these nanoparticles often increases due to aggregation.^[^ ^128^, ^129^ ^]^

Various types of protecting layers have been adopted to prevent aggregation that led to uniformly sized intermetallic nanoparticles upon annealing. SiO_2_‐coating^[^ ^130^ ^]^ and NaCl‐blending^[^ ^131^ ^]^ successfully protected PtFe nanoparticles from aggregation and were easily removed after annealing. Sun and co‐workers developed an effective stabilization strategy by coating random‐alloyed PtFe nanoparticles with MgO in benzyl ether, thereby maintaining a uniform size distribution after annealing to obtain ordered PtFe nanoparticles (Figure 9d).^[^ ^132^ ^]^ In a subsequent study, the authors employed this approach to obtain fully ordered PtFe nanoparticles supported on carbon by annealing MgO‐coated PtFe–Fe_3_O_4_ dumbbell‐shaped nanoparticles at 700 °C (5% H_2_ in Ar).^[^ ^133^ ^]^ The Hyeon group contributed to coating random‐alloy PtFe nanoparticles with polydopamine to in situ form N‐doped carbon shells during annealing. The N‐doped carbon coating protected the nanoparticles from coalescence while exposing the active catalytic surface of the intermetallic alloy nanoparticles toward the electrolyte without removing the shell (Figure 9e).^[^ ^134^ ^]^ Despite its less uniformity, the synthesis of intermetallic PtFe nanoparticles has recently been achieved by a wet‐chemical approach. Hou and co‐workers^[^ ^135^ ^]^ included halide ions (Cl^−^, Br^−^, or I^−^ by using NH_4_Cl, KBr, or KI, respectively) during the formation of PtFe nanoparticles in oleylamine so that the formation of the ordered L1_0_‐phase became thermodynamically favorable. The above‐mentioned strategies can readily be applied to the preparation of intermetallic Pt_3_Fe or PtFe_3_ nanoparticles of the L1_2_‐phase by controlling the atomic ratio between Pt and Fe in initial random‐alloy nanoparticles.^[^ ^21^, ^136^ ^]^

Interestingly, adding a third metal species to the Pt–Fe system to obtain doped or trimetallic PtFeM nanoparticles has been proven to promote atomic ordering and tune the strain of the Pt surface. Sun and co‐workers demonstrated that the addition of Au to the PtFe system facilitated the formation of the L1_0_ phase when annealed at 600 °C.^[^ ^137^ ^]^ The embedded gold atoms were segregated toward the surface and generated internal vacancies enabling the effective transformation of random‐alloy nanoparticles into intermetallic PtFe nanoparticles with Au‐enriched surfaces (Figure 9f). Wang and co‐workers introduced Ag into the wet‐chemical synthesis of L1_0_‐PtFe nanoparticles.^[^ ^138^ ^]^ By controlling the atomic percentage of Ag, they observed at a certain point (≈23 at%) Ag chemically segregates on PtFe with the simultaneous formation of an ordered L1_0_‐PtFe phase without a postannealing process, and the optimum point for the most significant ordering was at 29 at% of Ag. A trimetallic PtFeCu system with controllable atomic composition was systematically studied by Sun and co‐workers.^[^ ^139^ ^]^ Starting from presynthesized PtFe nanoparticles, a trimetallic solid solution was prepared by the reduction of Cu(acac)_2_ and diffusion of Cu into PtFe random alloy in 1‐octadecene and oleylamine at 240 °C. Although the inclusion of Cu in PtFe was detrimental for atomic ordering upon annealing, Cu played an important role in modulating the compressive strain applied to the L1_0_‐PtFe intermetallic nanoparticles, thus improving the catalytic activity. On the other hand, the substitution of 10% of Pt in Pt_3_Fe with Pd was recently found to promote atomic ordering in the L1_2_ phase through a simple impregnation method.^[^ ^140^ ^]^ The role of Pd was not limited to that, but also included the formation of PdH*_x_* to tune the electron density of the intermetallic nanoparticles.

Analogous to the Pt–Fe intermetallic system, there has been a great advancement in the synthesis of Pt–Co intermetallic nanoparticles. Although much attention has been paid to the importance of L1_0_‐PtCo or L1_2_‐Pt_3_Co nanoparticles for oxygen reduction electrocatalysis,^[^ ^141^, ^142^, ^143^ ^]^ precise synthesis of L1_0_‐PtCo nanoparticles on carbon support was recently realized by Sun and co‐workers (Figure 9g,h).^[^ ^144^ ^]^ Randomly alloyed A1‐PtCo (fcc) nanoparticles were prepared by thermal reduction of Pt(acac)_2_ and Co(acac)_2_ in oleylamine, which were loaded on a carbon support and annealed without any protecting layer. No sign of aggregation was found on the intermetallic nanoparticles owing to the low loading of 8 wt% and the high resistance of the PtCo system against aggregation. Similar to the case of PtFe, where Au was added to aid atomic ordering, PtCoAu nanoparticles with Au‐enriched L1_0_‐PtCo structure have been studied recently.^[^ ^145^ ^]^ The A1‐PtCoAu nanoparticles underwent annealing at 650 °C to be converted to the L1_0_ core and PtAu alloy shell of ≈2 atomic‐layer thickness. Furthermore, various trimetallic L1_0_‐PtCoM (M = Fe, Ni, Cu or Zn) nanoparticles were prepared by substituting half the amount of Co(acac)_2_ with M(acac)_2_ or M(acac)_3_ and adding a borane‐tert‐butylamine complex in the colloidal synthesis of random trimetallic alloys that underwent annealing.^[^ ^146^ ^]^ Li and co‐workers applied several dopants (W, Ga, or Zn) to L1_0_‐PtCo nanoparticles with a size of ≈3 nm by mixing a small amount of W(CO)_6_, Ga(NO_3_)_3_, or Zn(acac)_2_ with Co(acac)_2_ in the course of CoO shell formation on presynthesized Pt nanoparticles.^[^ ^147^ ^]^ After annealing the M‐Pt/CoO core–shell at 700 °C with MgO protection, the MgO shell on the intermetallic core was removed and the nanoparticles supported on carbon were further annealed at 400 °C without severe aggregation.

The intermetallic Pt–Ni system is less familiar than Pt–Fe and Pt–Co because of the low tendency of atomic ordering between Pt and Ni even with annealing. Early efforts on the synthesis of PtNi intermetallic nanomaterials have tried different metal precursors and reducing agents to develop a novel set of reagents.^[^ ^148^ ^]^ Reduction of Li_2_NiCl_4_ and Pt(COD)Cl_2_ (COD = cyclooctadiene) by potassium triethylborohydride (KBH(Et)_3_) produced small‐sized PtNi nanoparticles, which were converted to an intermetallic phase with severe aggregation by vacuum annealing at 500 °C. Li and co‐workers recently developed the synthesis of small‐sized L1_0_‐PtNi by annealing Pt/NiO*_x_* core/shell nanoparticles on a carbon support.^[^ ^149^ ^]^ The initially prepared ≈5 nm sized core/shell nanoparticles did not undergo severe aggregation upon annealing at 600 °C because of their fine dispersion on the carbon support. Sasaki and co‐workers introduced nitrogen into intermetallic PtNi nanoparticles by simply annealing carbon support, impregnated with Pt(acac)_2_ and Ni(acac)_2_, at 560 °C under an ammonia atmosphere.^[^ ^150^ ^]^ Despite the high Pt loading of 18.3 wt% in the final product, highly dispersed ≈4.7 nm L1_0_‐PtNiN nanoparticles were formed on the carbon support (Figure 9i,j).

The remaining 3d late transition metals, Cu and Zn, were alloyed with Pt in an atomically ordered way to produce L1_1_‐PtCu, L1_2_‐PtCu_3_, L1_2_‐Pt_3_Zn, and L1_0_‐PtZn. Intermetallic L1_1_‐PtCu has better electronic interaction between Pt and Cu than the L1_0_ or L1_2_ phases of Pt–Fe, Pt–Co, and Pt–Ni systems^[^ ^151^ ^]^ although it has mostly been developed recently with only a few reports.^[^ ^152^ ^]^ Xin and co‐workers prepared L1_1_‐PtCu nanoparticles on carbon by first impregnating Pt(acac)_2_ and Cu(acac)_2_ on carbon, followed by annealing at 800 °C under 5% H_2_ in Ar flow.^[^ ^153^ ^]^ Additionally, they doped nitrogen into the intermetallic PtCu alloy by flowing ammonia for 2 h at 500 °C before the temperature was raised to 800 °C (Figure 9k). Moreover, a similar impregnation method was also used to prepare L1_2_‐PtCu_3_ nanoparticles on carbon by Abruña and co‐workers through two‐step annealing in flowing H_2_.^[^ ^154^ ^]^ In the intermetallic PtZn nanoparticles, Murray and co‐workers reported monodisperse Pt_3_Zn random‐alloy nanoparticles by colloidal synthesis and annealed them at 600 °C to get L1_2_‐Pt_3_Zn nanoparticles with slight aggregation for the first time.^[^ ^155^ ^]^ Huang and co‐workers synthesized L1_0_‐PtZn nanoparticles on multiwalled carbon nanotubes (MWNTs), and small‐sized intermetallic nanoparticles less than 5 nm were obtained using a silica shell.^[^ ^156^ ^]^ The introduction of Zn into Pt/MWNT@mSiO_2_ was achieved by the thermal reduction of Zn(acac)_2_ in oleic acid and oleylamine at 330 °C, and the final intermetallic phase was obtained after annealing at 600 °C.

Other transition metals, such as Ti, V, Cr, Mn, and Sn, have been alloyed with Pt in an atomically ordered manner with limited control over their size, composition, and shape. The formation of L1_2_‐Pt_3_M nanoparticles of 3d early transition metals such as Ti,^[^ ^157^, ^158^ ^]^ V, ^[^ ^158^ ^]^ Cr,^[^ ^159^ ^]^ and Mn^[^ ^160^ ^]^ was achieved in a similar manner to those already described by annealing presynthesized colloidal random alloys with or without protecting agents. On the contrary, syntheses of small intermetallic PtSn (*P63/mmc*) and L1_2_‐Pt_3_Sn nanoparticles have been possible without further annealing,^[^ ^161^, ^162^ ^]^ although the formation of L1_2_‐Pt_3_Sn often requires a postannealing step.^[^ ^163^, ^164^ ^]^

Hard‐template and wet‐chemical approaches have mainly been employed to obtain shaped intermetallic nanoparticles. In a recent approach by Joo and co‐workers, for 1D intermetallic nanostructure of Pt_3_Co, mesoporous silica (SBA‐15) was infiltrated with a controlled amount of metal precursors followed by a typical annealing process under H_2_ flow.^[^ ^165^ ^]^ Although the HF washing step was necessary to remove the silica template after annealing, this hard‐template method uniquely provided a highly crystalline intermetallic Pt_3_Co phase with a controlled 1D morphology (Figure 9l,m). Moreover, the use of a 3D silica template (KIT‐6) also generated a regular 3D network of intermetallic Pt_3_Co by the same process. Less controlled but more efficient method to obtain 1D L1_2_‐Pt_3_Co nanowires was reported by Huang and co‐workers using an oil bath to simply reduce metal precursors in oleylamine in the presence of a surfactant (CTAC) and a reducing agent (glucose).^[^ ^166^ ^]^ The same group recently reported 1D L1_2_‐Pt_3_Sn through similar synthetic conditions by reducing metal precursors in oleylamine with a surfactant (stearyltrimethylammonium chloride, STAC) and Mo(CO)_6_.^[^ ^167^ ^]^ They controlled the aspect ratio of Pt_3_Sn nanofibers, from 22.5 to 13.4, with an average diameter of 1.7 nm, by changing the type of surfactant or solvent. 2D PtPb nanoplates with a hexagonal phase (*P63/mmc*) were also prepared similarly to 1D Pt‐intermetallics.^[^ ^168^ ^]^ Metal acetylacetonate precursors were reduced with oleylamine as a surfactant and ascorbic acid in octadecene. The initially formed Pb_3_(CO_3_)_2_(OH)_2_ transformed into intermetallic PtPb nanoplates by the reduction and interdiffusion of Pt. Quan and co‐workers recently discovered a facile wet‐chemical route to prepare trimetallic PtSnBi nanoplates (Figure 9n) with the same hcp structure (*P63/mmc*).^[^ ^169^ ^]^ Three different metal precursors were mixed and thermally decomposed in an oleylamine/octadecene mixture with a surfactant (CTAB) and ascorbic acid. The molar ratio of Sn to Bi was readily tuned by controlling the amount of metal precursors. As can be seen in the aforementioned works, where Sn and Pb can form intermetallic phases with Pt by wet‐chemical approaches, octahedron‐shaped PtPb (*P63/mmc*) with Ni doping^[^ ^170^ ^]^ and cube‐shaped L1_2_‐Pt_3_Sn^[^ ^171^ ^]^ nanoparticles were prepared by simply controlling the amount of ascorbic acid (PtPb) and via the hot‐injection method (Pt_3_Sn). For 3D intermetallic nanoparticles, Joo and co‐workers successfully synthesized very recently intermetallic L1_1_‐PtCu nanoframes without losing the 3D frame morphology by silica protection.^[^ ^151^ ^]^ The initially formed PtCu randomly alloyed nanoframes on a carbon support transformed into an ordered intermetallic state at 600 °C with a silica layer. The silica coating was then etched with HF, and the final form of the supported L1_1_‐PtCu nanoframe with a Pt‐skin layer was obtained after annealing at 300 °C.

**Figure 10:**
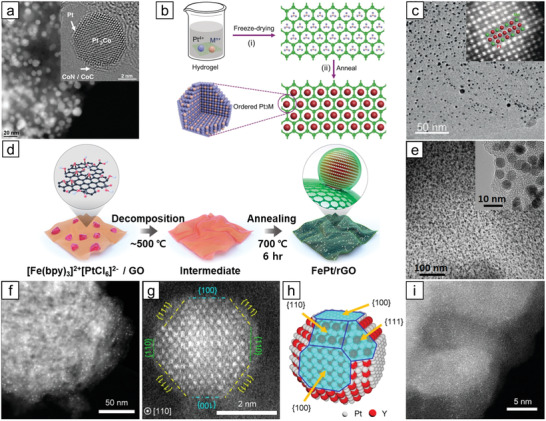
a) HAADF‐STEM image and high resolution TEM image (inset) of ZIF‐derived supported L1_2_‐Pt_3_Co nanoparticles. Reproduced with permission.^[^ ^174^ ^]^ Copyright 2018, AAAS. b) Schematic illustration of the synthesis of L1_2_‐Pt_3_M nanoparticles on rGO by annealing and c) TEM image of the prepared Pt_3_Mn/rGO sample with STEM image (inset). Reproduced with permission.^[^ ^181^ ^]^ Copyright 2020, Wiley‐VCH. d) Schematic illustration of the synthesis of L1_0_‐PtFe nanoparticles on rGO by decomposition of bimetallic compound and e) TEM images of PtFe/rGO. Reproduced with permission.^[^ ^182^ ^]^ Copyright 2020, American Chemical Society. f,g) HAADF‐STEM images of highly dispersed Pt_3_Y nanoparticles on mesoporous zeolite, h) their proposed atomic arrangement, and i) HAADF‐STEM image of atomically dispersed La atoms by silanol nests of zeolite. Reproduced with permission.^[^ ^184^ ^]^ Copyright 2020, The Authors, under exclusive licence to Springer Nature Limited.

Lastly, recent novel approaches using the heat‐induced formation of Pt‐intermetallic nanoparticles have garnered attention due to several advantages of simultaneous formation of nanoparticles and their atomic ordering in a single annealing step, along with high potential scalability for practical applications. One important advancement is the application of zeolitic imidazolate framework (ZIF)‐derived carbon (ZIF‐C) as a source of both carbon support and transition metals.^[^ ^172^, ^173^, ^174^ ^]^ Similar kinds of ZIF materials, composed of Co and Zn metals, each coordinated by four imidazolate groups, were first pyrolyzed above 700 °C to obtain highly porous carbons in the presence of Co. After loading a certain amount of Pt nanoparticles on them, an additional annealing process was applied to form intermetallic L1_0_‐PtCo^[^ ^172^, ^174^ ^]^ or L1_2_‐Pt_3_Co^[^ ^173^, ^174^ ^]^ nanoparticles on ZIF‐C (**Figure** **10**a).

A significant but more facile approach that can directly convert well‐defined metal precursors into multimetallic nanoparticles has recently attracted a lot of attention because of the enhanced uniformity in size and composition compared to conventional impregnation methods.^[^ ^175^, ^176^ ^]^ Even though the overall synthetic procedures are often simple, the precise design of the structure of multimetallic precursors itself or on support is critical to the product obtained after annealing.^[^ ^177^, ^178^, ^179^, ^180^ ^]^ Cui and co‐workers provided a general strategy to prepare Pt‐intermetallic nanoparticles on reduced graphene oxide (rGO) by impregnating metal precursors on GO in the presence of poly(vinyl alcohol) (PVA). PVA forms hydrogen bonds with GO and ensures uniform coating of metal precursors on it before freeze‐drying and annealing at 700 °C for 12 h to obtain ultrasmall (≈3 nm) L1_2_‐Pt_3_M (M = Cr, Mn, Fe, Co) nanoparticles on rGO (Figure 10b,c).^[^ ^181^ ^]^ The Hyeon group recently designed a synthetic concept to prepare intermetallic Pt nanoparticles with 1:1 composition by uniform decomposition of bimetallic compounds with the formula [M(bpy)_3_]^2+^[PtCl_6_]^2−^ (M = Fe, Co, or Ni; bpy = 2,2′‐bipyridine).^[^ ^182^ ^]^ Bimetallic compounds with sizes of several hundreds of nanometers, simply prepared by mixing separate solutions of M^2+^, bpy, and PtCl_6_^2−^, were each attached to GO and freeze‐dried. The composite underwent thermal annealing up to 700 °C, during which the surface of GO was uniformly coated with a thin layer of decomposed bimetallic compounds (≈500 °C) and intermetallic nanoparticles grew while confined in the in situ formed N‐doped carbon shell surrounding them (Figure 10d,e). Despite the 6 h annealing period at 700 °C, the L1_0_‐PtFe, L1_0_‐PtCo, and PtNi nanoparticles maintained their uniform sizes, while the constituent metal atoms migrated enough to form intermetallic phases (for PtFe and PtCo).

Among many efforts to obtain intermetallic Pt‐alloy nanoparticles with low reduction potential metals,^[^ ^183^ ^]^ Ryoo and co‐workers successfully prepared highly dispersed and atomically ordered L1_2_‐Pt_3_Y or L1_2_‐Pt_3_La nanoparticles in mesoporous zeolite by creating silanol nests (through degallation of mesoporous gallosilicate zeolite) that could anchor platinum and rare‐earth elements (Y or La) as single atomic species upon impregnation (Figure 10f–h).^[^ ^184^ ^]^ The atomic dispersion of Y and La, as confirmed by HAADF‐STEM images, was considered to be critical for their diffusion into Pt and the formation of intermetallic phases despite their low chemical reduction potential (Figure 10i). In summary, the above‐mentioned recent references demonstrate that novel approaches to thermally control the nucleation and growth processes of intermetallic nanoparticles are now actively being discussed and provide valuable insights to obtain desirable nanomaterials (whether uniform or uncommon structures) via simple and scalable methods.

#### 2.4.2 Pd Intermetallics

**Figure 11:**
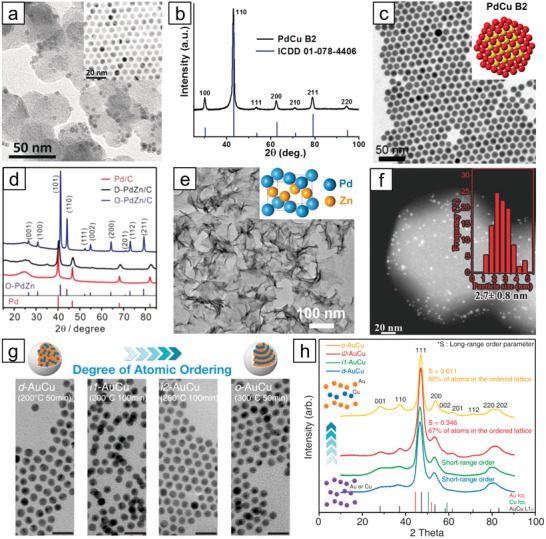
a) TEM images of B2‐PdCu/C and A1‐PdCu nanoparticles (inset). Reproduced with permission.^[^ ^186^ ^]^ Copyright 2020, Wiley‐VCH. b) XRD pattern of B2‐PdCu nanoparticles prepared from A1‐PdCu seeds and c) their TEM image with proposed atomic arrangement (inset). Reproduced with permission.^[^ ^187^ ^]^ Copyright 2016, American Chemical Society. d) XRD pattern of L1_0_‐PdZn phase obtained by impregnation method. Reproduced with permission.^[^ ^188^ ^]^ Copyright 2019, American Chemical Society. e) TEM image of L1_0_‐PdZn nanosheets with proposed atomic arrangement (inset). Reproduced with permission.^[^ ^189^ ^]^ Copyright 2019, American Chemical Society. f) HAADF‐STEM image of supported L1_0_‐PdZn nanoparticles on ZIF‐C. Reproduced with permission.^[^ ^190^ ^]^ Copyright 2018, Wiley‐VCH. g) TEM images and h) XRD patterns of AuCu nanoparticles with tuned degree of ordering. Reproduced with permission.^[^ ^204^ ^]^ Copyright 2017, American Chemical Society.

Unlike Pt‐intermetallics, the domain of ordered Pd‐intermetallic nanoparticles has been quite limited until recently. The most studied category is the Pd–Cu system, as the B2‐PdCu (body‐centered cubic) phase is readily formed by annealing below 400 °C. Synthesis of monodisperse A1‐PdCu nanoparticles with sizes of ≈5 nm was carried out in an oil bath by Huang and co‐workers.^[^ ^185^, ^186^ ^]^ After annealing at 350 °C (or 375 °C), the A1‐PdCu nanoparticles evenly distributed on a carbon support were transformed into ordered B2‐PdCu without aggregation (**Figure** **11**a,b). They also added a third metal species such as Co or Ni (of about 20 at% in trimetallic alloy) into the PdCu system and obtained monodisperse B2‐PdCuM (M = Co or Ni) nanoparticles on carbon after annealing.^[^ ^185^ ^]^ Skrabalak and co‐workers successfully synthesized B2‐PdCu via a wet‐chemical approach that produced monodisperse B2‐PdCu nanoparticles from the co‐reduction of PdBr_2_ and Cu(ac)_2_ (ac: acetate) at 235 °C on presynthesized disordered A1‐PdCu seeds (Figure 11c).^[^ ^187^ ^]^ The authors attributed the phase transition from A1 to B2 to the increase of size from 6.8 nm (of A1‐PdCu seeds) to 11.9 nm (B2‐PdCu nanoparticles), which made each nanoparticle overcome the activation barrier for atomic ordering. In the case of PdZn, an intermetallic phase is manifested as L1_0_‐PdZn, which can be simply prepared at 500 °C by impregnation method (Figure 11d).^[^ ^188^ ^]^ Zhang and co‐workers reported the colloidal synthesis of nearly spherical L1_0_‐PdZn nanoparticles and nanosheets of L1_0_‐PdZn, L1_0_‐PdCd, and L1_0_‐PdZnCd phases using Mo(CO)_6_ (Figure 11e).^[^ ^189^ ^]^ Metal acetylacetonate precursors were coreduced at 325 °C in the presence of Mo(CO)_6_, which produced atomically ordered nanosheets with thicknesses of less than 5 nm. Moreover, Chen and co‐workers recently adopted a simple impregnation method on ZIF‐C to prepare sub‐2 nm L1_0_‐PdZn nanoparticles after annealing at 400 °C (Figure 11f).^[^ ^190^ ^]^ The ZIF‐C (obtained by calcining ZIF‐8 at 650 °C) was impregnated with controlled amounts of Pd and Zn precursors to obtain 1.2, 1.8, 2.7, and 10 nm‐sized ordered PdZn nanoparticles with restricted aggregation due to the rich porous structure of ZIF‐C.

Similar to several reports on Pt intermetallic nanoparticles,^[^ ^147^, ^149^ ^]^ core–shell Pd@FeO*_x_* nanoparticles were also prepared for conversion into ordered L1_0_‐PdFe nanoparticles of uniform sizes by annealing at 600 °C on a carbon support.^[^ ^191^, ^192^ ^]^ Moreover, carbon‐supported L1_2_‐Pd_3_Fe nanoparticles were directly synthesized by heat‐induced formation from KCl‐protected precursors on carbon^[^ ^193^ ^]^ or cyanogel‐coated carbon.^[^ ^194^ ^]^ On the contrary, Pd–Ni or Pd–Co systems are much less known than those already described because of the greater difficulty in the formation of an intermetallic phase through annealing.^[^ ^195^ ^]^ On the other hand, L1_2_‐Pd_3_Pb nanomaterials have been recently developed via facile oil bath methods, providing nanoplates and 3D nanowire networks.^[^ ^196^, ^197^ ^]^ Finally, the intermetallic Pd–Bi system covers a wide range of ordered phases; however, control over size and shape has been largely limited.^[^ ^198^ ^]^ Recent efforts on this highly complicated system have offered supported nanoparticles of different intermetallic phases, such as Pd_3_Bi nanoparticles, by electrochemical dealloying of carbon‐supported *β*‐PdBi_2_,^[^ ^199^ ^]^ *β*‐PdBi nanoparticles prepared via impregnation reduction at 600 °C,^[^ ^200^ ^]^ and Pd_31_Bi_12_ nanoparticles electrochemically deposited on carbon support at room temperature.^[^ ^201^ ^]^

#### 2.4.3 Au Intermetallics

The scope of Au intermetallic nanomaterials is narrower than that of Pt and Pd intermetallic nanomaterials. Early works by Schaak and co‐workers provided valuable insights into the formation of L1_0_‐AuCu and L1_2_‐AuCu_3_ nanoparticles.^[^ ^202^, ^203^ ^]^ Although the obtained nanoparticles were polydisperse and irregularly formed, they found that the ordered L1_0_‐AuCu phase starts to nucleate at 200 °C, and the phase is maintained between 200 and 400 °C upon annealing in Ar atmosphere, while the L1_2_‐AuCu_3_ phase nucleates at ≈300 °C by diffusion of Cu into the AuCu intermediate.^[^ ^202^ ^]^ Thereafter, they reported a wet‐chemical approach for the preparation of L1_0_‐AuCu and L1_2_‐AuCu_3_ nanoparticles and nanowire networks by using polyols to reach up to 310 °C.^[^ ^203^ ^]^ Recent works successfully prepared monodisperse L1_0_‐AuCu via seed‐mediated growth in wet‐chemical systems using oleic acid or oleylamine. Yang and co‐workers^[^ ^204^ ^]^ tuned the degree of ordering in an AuCu nanoparticle by controlling the temperature (200–300 °C) and time (50–100 min), while Zhu and co‐workers^[^ ^205^ ^]^ obtained disordered and ordered AuCu nanoparticles by controlling the temperature (210 or 290 °C), both starting from presynthesized Au seed nanoparticles (Figure 11g,h). Another recent work published by Skrabalak and co‐workers started from disordered A1‐AuCu nanoparticles to prepare monodisperse L1_0_‐AuCu at 280 °C with an increase in size from 6.5 to 10.3 nm.^[^ ^206^ ^]^ The seed‐mediated approach was also used to prepare intermetallic AuSn (NiAs‐type) and AuSn_2_ (orthorhombic) nanoparticles via the diffusion of Sn into presynthesized Au nanoparticles upon hydride reduction.^[^ ^207^, ^208^ ^]^ By controlling the amount of Sn precursor, single‐phased or multiphased AuSn/AuSn_2_ intermetallic nanoparticles could be readily prepared. Other Au intermetallic nanoparticles such as *β*′‐AuZn, R1‐Au_3_Zn,^[^ ^209^ ^]^ L1_2_‐Au_3_M (M = Fe, Co or Ni),^[^ ^210^ ^]^ and L1_2_‐Au_3_Cu^[^ ^211^ ^]^ have been possible through colloidal syntheses at temperatures between 200 and 250 °C, mainly developed by Schaak and co‐workers.

### 2.5 Core–Shell Structures

#### 2.5.1 Seed‐Mediated Synthesis

**Figure 12:**
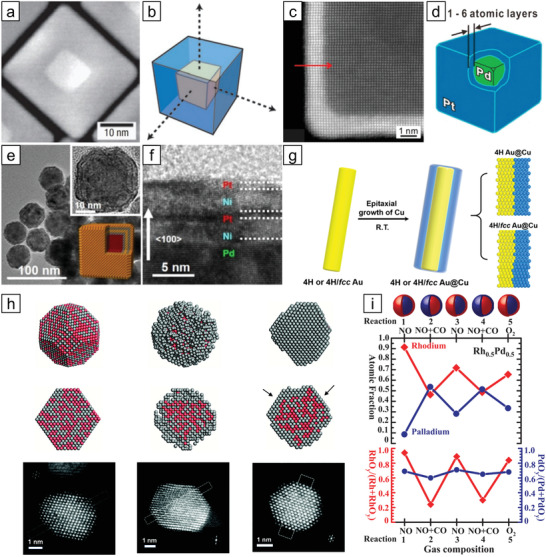
a) HAADF‐STEM image of Pt@Pd nanocubes and b) a schematic illustration of the morphology. Reproduced with permission.^[^ ^213^ ^]^ Copyright 2007, Nature Publishing Group. c) HAADF‐STEM image of a Pd@Pt nanoparticle and d) a schematic illustration of control of shell thickness. Reproduced with permission.^[^ ^220^ ^]^ Copyright 2014, American Chemical Society. e) TEM images and the proposed structure of Pd–(Ni–Pt)_2_ nanoparticles and f) their magnified TEM image. Reproduced with permission.^[^ ^242^ ^]^ Copyright 2020, Wiley‐VCH. g) Schematic illustration of epitaxial Cu shell formation on 4H (or 4H/fcc) Au nanorods. Reproduced with permission.^[^ ^245^ ^]^ Copyright 2020, American Chemical Society. h) Schematic diagram (up) and HAADF‐STEM images (down) of pristine (left), acid‐etched (center, Pt‐skeleton), and thermally annealed (right, Pt‐skin) PtNi catalysts. Reproduced with permission.^[^ ^259^ ^]^ Copyright 2011, American Chemical Society. i) Reversible change in surface composition of RhPd nanoparticles under different gas environments. Reproduced with permission.^[^ ^267^ ^]^ Copyright 2008, AAAS.

The core–shell structure has gained much attention in electrocatalysis due to its ability to precisely tune the surface properties of active metal species by the core and the reduced amount of precious noble metals used, which contributes to higher mass activity. Until recently, most of the works that produced novel core–shell nanoparticles with elaborate control over morphologies relied on a seed‐mediated approach.^[^ ^212^ ^]^ Heterogeneous nucleation of secondary metal species on seeds is important to obtain only core–shell products, which is typically achieved by low concentrations of secondary metal precursors during shell growth. The epitaxial growth of secondary metal shells usually follows the atomic arrangements on specific facets of core nanocrystals, thereby retaining the initial morphologies of the cores (**Figure** **12**a,b).^[^ ^213^, ^214^ ^]^ The two well‐known combinations of metals are Pt–Pd and Au–Ag, as they both have lattice mismatches of less than 1%.^[^ ^213^, ^214^, ^215^, ^216^ ^]^ The greater the lattice mismatch between two metal elements, the lower the likelihood of shell formation and the poorer control over shell thickness, which has been identified in efforts to deposit 3d transition metals on Pd or Au seeds.^[^ ^217^, ^218^, ^219^ ^]^

Owing to the low to moderate lattice mismatches with Pd, the shells of Pt, Ir, Rh, and Ru have been prepared on various structures of Pd nanoparticle seeds with a controlled number of shell atomic layers. Layer‐by‐layer conformal deposition of Pt atomic layers on Pd nanocubes was reported in 2014 by the slow injection rate of Pt at 200 °C, which allowed enough time and energy for Pt atoms to spread onto the entire surface of Pd (Figure 12c,d).^[^ ^220^ ^]^ The same synthetic concept has been adopted with little modification to deposit several atomic layers of Pt shells on Pd nanocrystals with twinned decahedral,^[^ ^221^ ^]^ twinned icosahedral,^[^ ^222^ ^]^ and octahedral^[^ ^223^ ^]^ shapes. Reaction kinetics have also been controlled recently by the amount of reductant (glucose) for the deposition of a uniform shell or islands of Pt on Pd nanocubes.^[^ ^224^ ^]^ Moreover, Qiu and co‐workers recently provided tetrahedral core–shell nanoparticles by deposition of Pt, Ru, or Rh on tetrahedral Pd seeds, obtaining less than five atomic layers of shells.^[^ ^225^ ^]^ For deposition of Ir shells on Pd nanoparticles, Xia and co‐workers reported layer‐by‐layer deposition of Ir shells rather than island formation through slow injection of Ir solution into a mixture of reagents containing ethylene glycol, PVP, ascorbic acid, and Pd seeds (cubes or octahedra).^[^ ^226^ ^]^ Recently, the method was used to precisely control the number of atomic layers of the Ir shell and correlated it with the electrocatalytic activity and durability.^[^ ^227^ ^]^ Although Ag has a large lattice mismatch of ≈4.5% with Pd, Xia and co‐workers successfully prepared Ag shells on Pd nanocubes by a fast reduction kinetics, which produced Ag coating within 2 min.^[^ ^228^ ^]^ The authors attributed the anomalous shell formation to the small size of Pd seeds (18 nm), which enabled nucleation and epitaxial growth of Ag shells on the {100} facets. On the other hand, deposition of Au on Pd seeds generally forms PdAu shells of a few atomic layers by galvanic replacement.^[^ ^229^, ^230^ ^]^

Another widely used core is Au, which usually promotes durability against electrochemical leaching, thereby providing unique stability to the shell.^[^ ^231^ ^]^ The deposition of Pd^[^ ^232^, ^233^ ^]^ or Pt^[^ ^233^, ^234^ ^]^ shells on Au seeds was carried out under mild conditions in which the addition of Pd or Pt precursor solution and reductant resulted in uniform shells on Au. Furthermore, Sun and co‐workers have reported the synthesis of alloy shells of various compositions, such as PtCu^[^ ^235^ ^]^ and PdCu,^[^ ^236^ ^]^ simply by placing Au seeds before coreduction of two metal precursors with controlled atomic ratios. This approach has enabled the production of various alloy shells on Au or Pd cores such as Au@PtFe,^[^ ^237^ ^]^ Au@PtNi,^[^ ^238^ ^]^ Pd@PtFe,^[^ ^239^ ^]^ Pd@Au@PtFe,^[^ ^240^ ^]^ and Pd@PdFe core@shell nanoparticles.^[^ ^241^ ^]^ Recently, Tsung and co‐workers reported an effective strategy to prepare intermetallic alloy shells on Pd cores through thermal intermixing of Pt and Ni from epitaxially grown Pt and Ni shells.^[^ ^242^ ^]^ Compared to thicker Ni–Pt shells on Pd, the 4‐layered Pd–(Ni–Pt)_2_ (Pd–(Ni–Pt–Ni–Pt)) core–shell structure (Figure 12e,f) induced strain and provided a shorter diffusion length between Pt and Ni, so that only 400 °C was sufficient for full mixing of Pt and Ni to produce intermetallic PtNi_3_ shells on Pd nanocubes, while 2‐layered Pd–(Ni–Pt) with thicker shells of Pt and Ni showed incomplete intermixing after annealing. Finally, epitaxial shell growth on unconventional phases has recently been explored by Zhang and co‐workers, starting from 4H/fcc Au nanorods, 2H Pd nanoparticles, and 4H(or 4H/fcc) Au nanorods to prepare 4H/fcc Au@Pd,^[^ ^243^ ^]^ fcc‐2H‐fcc Pd@M (M = Au, Ag, or Pt),^[^ ^244^ ^]^ and 4H(or 4H/fcc) Au@Cu nanoparticles (Figure 12g)^[^ ^245^ ^]^ under mild conditions, which are already well known for shell growth.

#### 2.5.2 One‐Step Synthesis

As discussed in Section 2.1.5, a huge gap between the reduction rates of the constituent metals leads to phase‐segregated nanoparticles. As Au@Pd nanoparticles have a large gap in reduction potential, they have been frequently demonstrated as representative examples for the one‐step synthesis of core–shell structures. In 2009, Han and co‐workers reported the one‐step synthesis of Au@Pd via the coreduction of HAuCl_4_ and K_2_PdCl_4_ in the presence of CTAC.^[^ ^246^ ^]^ Temporal investigation of the metal ratio in a nanoparticle indicated that the formation of Au octahedra takes place first, and a Pd layer forms on them due to the huge difference in the reduction rates of the metal precursors. In a control experiment, the use of ascorbic acid, which is a stronger reductant, resulted in AuPd alloy nanoparticles, owing to the rapid reduction of the precursors. The synthesis of other core–shell nanoparticles with various combinations of metals, such as Rh@Fe^[^ ^247^ ^]^ and Cu@Ir^[^ ^248^ ^]^ Pd@PdPt,^[^ ^249^ ^]^ could also be achieved via this one‐pot approach through appropriate selection of chemicals and reaction conditions. A recent study by Shao and co‐workers attempted to control the shell thickness by varying the ratio of metal precursors, although one‐pot synthesis provides less control over the thickness and composition of shells than the seed‐mediated approach.^[^ ^250^ ^]^

The one‐pot synthesis of a more complex core–shell structure was demonstrated by the use of IrCl_3_ and Ir(acac)_3_, which are known to have quite different reduction potentials. Zhuang and co‐workers took advantage of the dual precursor approach to produce IrNi@Ir alloy core–shell nanoparticles.^[^ ^251^ ^]^ The synthesis proceeded by the simultaneous reduction of Ir(acac)_3_ and Ni(acac)_2_ to form an alloy core, followed by successive reduction of IrCl_3_ on the surface of the IrNi alloy, forming an Ir shell. Likewise, Lee and co‐workers synthesized alloy core@alloy shell nanoparticles by reducing dual Ir precursors (Ir(acac)_3_ and IrCl_3_) and dual transition (Ni and Cu) metal precursors to obtain double‐layered nanoframes.^[^ ^252^ ^]^ When only one Ir precursor, Ir(acac)_3_ or IrCl_3_, was used, only single‐layered nanoframes were produced.

#### 2.5.3 Transformation from Alloy

Apart from the afore‐mentioned approaches, the core–shell structure can be derived from alloy structures. Owing to the lower reduction tendency of non‐noble metals than noble metals, they are relatively vulnerable to chemical/electrochemical leaching under corrosive conditions. This difference in intrinsic stability provides another opportunity for the preparation of core–shell nanoparticles by dealloying the surface of alloy nanoparticles via chemical and electrochemical etching.

In recent years, the dealloying route has been intensively investigated for the preparation of noble metal‐based core–shell nanomaterials. In particular, nanostructures with Pt‐rich shells have attracted enormous interest owing to their greatly enhanced activity and stability toward oxygen reduction catalysis.^[^ ^253^, ^254^ ^]^ Chemical, electrochemical, or even combined etching has been conducted to dealloy the surface of Pt‐based random alloys, such as PtCu,^[^ ^154^, ^255^, ^256^ ^]^ PtCo,^[^ ^257^ ^]^ PtNi,^[^ ^258^, ^259^, ^260^, ^261^ ^]^ PtAg,^[^ ^262^ ^]^ and PtCuCo,^[^ ^263^ ^]^ to produce Pt‐rich shells on Pt‐alloy cores. Recently, this approach has been further enhanced by the use of additional thermal treatment after selective dealloying. The resultant structural transformation from the Pt skeleton to the Pt‐skin surface^[^ ^257^, ^259^ ^]^ (Figure 12h) altered the sorption properties by reducing the low‐coordinated surface sites, resulting in enhanced activity and durability. The post‐treatment of intermetallic nanoparticles recently produced L1_0_‐FePt@Pt^[^ ^264^ ^]^ and L1_0_‐CoPt@Pt^[^ ^144^ ^]^ from the corresponding intermetallic alloys.

In addition to Pt‐based materials, the synthesis of core–shell nanoparticles based on other noble metals, such as Ir and Au, has also been investigated for specific electrochemical reactions. IrNi@IrO*_x_* core–shell nanoparticles were prepared using two different dealloying procedures.^[^ ^265^ ^]^ The formation of IrO*_x_* on the surface was promoted either by sequential dealloying and oxidation or by coupled dealloying/oxidation. AuNi/Au core–shell nanoparticles were also produced by electrochemical dealloying of AuNi nanoparticles.^[^ ^266^ ^]^

Another approach to obtain core–shell structures from alloys is thermodynamically driven phase segregation by reducing the surface energy. Phase segregation from homogeneously alloyed nanoparticles can be induced in the presence of proper surface adsorbates because of their different binding affinities for each metal atom. Somorjai and co‐workers conducted a model study using Rh_0.5_Pd_0.5_ and Pt_0.5_Pd_0.5_ nanoparticles to investigate the effect of binding affinity between gases and metals on the elemental distribution in a nanoparticle.^[^ ^267^ ^]^ As the composition of gases changed from oxidizing to reducing conditions, the richness of metal on the surface was reversed in the case of Rh_0.5_Pd_0.5_, implying that the gaseous environment is important for the elemental distribution of metal atoms in a particle (Figure 12i). On the other hand, the annealing temperature also affected the surface property of products with a constant environment.^[^ ^268^ ^]^ Similar to the above studies, the PdCo@Pd core–shell structure could be induced by thermal annealing of PdCo alloy nanoparticles under H_2_ flow.^[^ ^269^ ^]^ The absorbate‐induced transformation can also be achieved by an electrochemical process.^[^ ^270^ ^]^ PtCo@Pt core–shell nanoparticles were fabricated via electrochemical annealing of PtCo alloy nanoparticles in a CO‐saturated electrolyte for an hour. Owing to the stronger binding of CO on Pt than Co, the surface of the nanoparticles was enriched by Pt, forming PtCo@Pt core–shell nanoparticles.

### 2.6 Other Heterostructures

**Figure 13:**
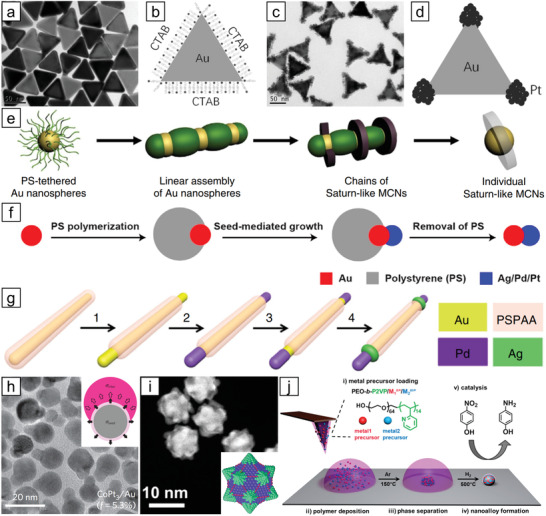
TEM images and schematic illustrations of a,b) Au nanotriangles and c,d) Au–Pt heterostructure. Reproduced with permission.^[^ ^275^ ^]^ Copyright 2016, Wiley‐VCH. Schematic illustrations of the polymer‐mediated preparation of e) coaxial and f) dimeric heterostructures. e) Reproduced under the terms of the Creative Commons CC BY license.^[^ ^276^ ^]^ Copyright 2016, The Authors. Published by Springer Nature. f) Reproduced with permission.^[^ ^277^ ^]^ Copyright 2019, American Chemical Society. g) Schematic illustration of the sequential deposition of metals on exposed Au surface after sequential removal of polymeric shell. Reproduced under the terms of the Creative Commons CC BY license.^[^ ^278^ ^]^ Copyright 2018, The Authors. Published by Springer Nature. h) TEM image of Au grown on Pt_3_Co seeds and a schematic illustration of applied stresses (inset). Reproduced with permission.^[^ ^280^ ^]^ Copyright 2014, Nature Publishing Group. i) HAADF‐STEM image with proposed structural image of Pd–Pt icosahedra. Reproduced with permission.^[^ ^286^ ^]^ Copyright 2021, American Chemical Society. j) Schematic illustration of SPBCL technique to prepare various heterostructured nanoparticles. Reproduced with permission.^[^ ^288^ ^]^ Copyright 2015, American Chemical Society.

Unlike alloys that contain more than two different metal elements in a single phase, the heterostructure features separate domains of metal elements. Most attempts to successfully obtain well‐defined and fine‐tuned heterostructured nanoparticles have relied on seed‐mediated syntheses. The large lattice mismatches between Au and 3d transition metals can easily deliver Au‐M dimers by the deposition of Cu or Fe on single‐crystalline Au seeds.^[^ ^271^, ^272^ ^]^ However, because of the similar atomic radii of Ag and Au, anisotropic growth of Ag on Au core has been achieved by the use of twinned Au nanoparticles (e.g., decahedron),^[^ ^273^ ^]^ which also proved effective for obtaining Au–Cu dimer nanoparticles through Cu deposition on twinned icosahedral Au seeds rather than single‐crystalline Au seeds.^[^ ^274^ ^]^ On the other hand, Wang and co‐workers employed an irregular distribution of surfactant molecules on shaped nanoparticles to selectively deposit Pt at the tips of Au nanotriangles, where fewer CTAB molecules were covered (**Figure** **13**a–d).^[^ ^275^ ^]^ Recently, for the deposition of noble metals on Au seeds, polymeric materials were deposited on Au to cover certain portions of the surface, followed by selective loading of other noble metals (Pd, Pt, or Ag) to form coaxial^[^ ^276^ ^]^ or dimeric^[^ ^277^ ^]^ heterostructures (Figure 13e,f). Furthermore, Chen and co‐workers precisely controlled how polymeric shells cover the surface of Au nanoparticles by forming polystyrene‐block‐poly(acrylic acid) shells on Au in the presence of different types of ligands and their heat‐induced transformation.^[^ ^278^ ^]^ Pd and Ag were sequentially deposited on Au, covering only the exposed surface of the Au seeds after the polymeric shells were thermally contracted or dissociated (Figure 13g).

Similarly, various heterostructured nanoparticles have been prepared from Pt or Pd seeds. The anisotropic growth of Au on Pt is well established because of their large lattice mismatch, while the deposition of Pd results in an epitaxial shell on the Pt core.^[^ ^213^ ^]^ Shevchenko and co‐workers reported a general nucleation and growth model of Au on Pt or Pt‐alloy seeds (Pt_3_Co or PtFe) by controlling the type of Au precursor (AuCl and AuCl_3_) and the use of foreign ions (Co^2+^ or Pb^2+^). They observed that Au^+^ ions are essential for the initial nucleation of Au on Pt, and the foreign ions promote the reduction of Au^3+^ to Au^+^ for nucleation.^[^ ^279^ ^]^ Further, they systematically uncovered three distinct periods for the formation of seed/Au dumbbell structures, which comprise prenucleation, nucleation, and growth.^[^ ^280^ ^]^ Surprisingly in the prenucleation period, Au formed an epitaxial shell on the seed, exerting lattice expansion on the seed by high stress of ≈2.4 GPa. A strain‐free domain of Au developed after the prenucleation period to relieve the lattice strain on the seed, leading to heterostructured dumbbell nanoparticles (Figure 13h). Notwithstanding the similar lattice parameters of Pt and Pd, avoiding shell formation by localized epitaxial growth of Pd on Pt seeds was demonstrated by increasing the reduction rate under high pH conditions (pH ≈ 9).^[^ ^281^ ^]^ Likewise, dendritic Rh nanostructures, rich in (100) facets, on Pt nanocubes were prepared by thermal decomposition of Rh(acac)_3_ at 200 and 170 °C, even in epitaxial growth.^[^ ^282^ ^]^

The formation of Pt branches on Pd nanocrystals was accomplished by the reduction of K_2_PtCl_4_ using ascorbic acid in the presence of Pd seeds.^[^ ^283^ ^]^ A more direct route to obtain an analogous structure was subsequently reported, which reduced both K_2_PtCl_4_ and Na_2_PdCl_4_ at 30 °C using Pluronic P123 as a structural template.^[^ ^284^ ^]^ Xia and co‐workers elaborated the seeded growth of Pt on Pd by introducing Br^−^ ions to transform PtCl_6_^2−^ (or PtCl_4_^2−^) to PtBr_6_^2−^ (or PtBr_4_^2−^), which promoted selective galvanic replacement on the vertices of Pd nanocubes^[^ ^285^ ^]^ and icosahedra by slow reduction kinetics (Figure 13i).^[^ ^286^ ^]^ Manipulation of reaction kinetics also played a key role in controlling the nucleation and growth of Ag or Au on Pd nanocubes, enabling growth at only one facet at a slow injection rate of Ag (or Au) and complete coverage in one‐shot injection.^[^ ^228^, ^287^ ^]^

Lastly, heat‐induced formation of heterostructured nanoparticles on a substrate has been actively studied by Mirkin and co‐workers using a technique called scanning‐probe block copolymer lithography (SPBCL).^[^ ^288^ ^]^ They used atomic force microscopy probes to transfer controlled amounts of polymer inks composed of poly(ethylene oxide)‐block‐poly(2‐vinylpyridine) and combinations of metal precursors. Upon two‐step thermal annealing under Ar and H_2_ flow, metal precursors were first aggregated and then reduced to a single multimetallic nanoparticle for each polymer dome on a substrate (Figure 13j). When the metal compositions of polymer inks were chosen to combine immiscible metals, heterostructured nanoparticles formed after annealing such as AuCo, AuNi, or AgCuCo.^[^ ^111^ ^]^ They further extended to study how Pd–Sn alloy system interacts with other multiple additional metals (Au, Ag, Co, Ni, or Cu) to determine design rules to modulate the number and types of phase boundaries in polyelemental nanoparticles with different tunable compositions.^[^ ^289^ ^]^ A recent study discussed a library of heterostructured nanoparticles with HIFs by the presence of Bi powders upstream of the tube furnace during the annealing of spin‐coated metal salts.^[^ ^290^ ^]^ They reported uniform heterostructured nanoparticles of HIFs using the SPBCL technique.

## 3 Considerations for Electrocatalytic Applications of NMMNs

**Figure 14:**
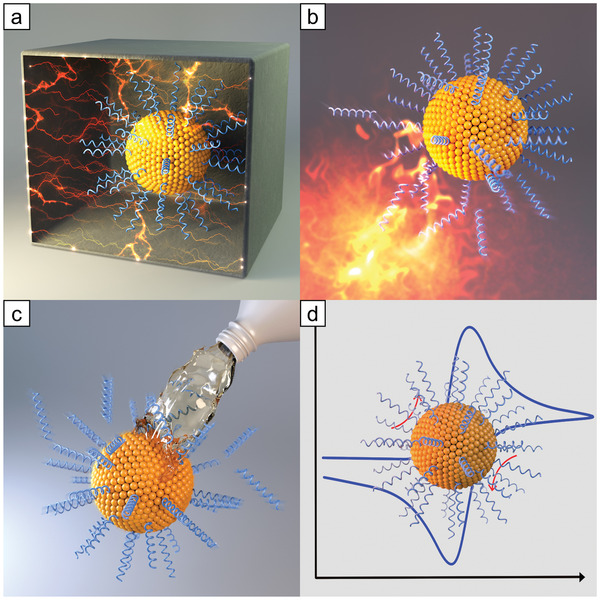
Representative strategies to remove surfactants for electrochemical applications. a) UVO treatment, b) thermal annealing, c) chemical treatment (acid treatment and ligand exchange), and d) electrochemical method.

As discussed in earlier sections, the designed synthesis of nanoparticles has achieved great success in controlling the size, shape, composition, and multifunctionalities of nanoparticles. However, several points remain to be considered for effective electrochemical energy conversion applications. To begin with, although surfactants (ligands) play pivotal roles in precise control over the structure and prevention of agglomeration during the synthesis of NMMNs, they hinder surface reactions. Covering the surface with surfactants hinders the effective utilization of principal active sites on the surface of nanoparticles, leading to poor catalytic performance. Therefore, it is necessary to establish efficient approaches to remove or tune the state of the surfactants.^[^ ^291^, ^292^ ^]^ It is necessary to carefully consider whether the original structural features of nanoparticles, such as facets, composition, strain, and intended functionalities, can be maintained after the removal processes. Second, we should consider how to deposit and stabilize NMMNs on support materials for the efficient utilization of prepared nanoparticles in electrocatalytic applications. In order to overcome the intrinsic instability of nanoparticles and to maximize their surface utilization, their dispersion on solid supports has been extensively investigated in the field of heterogeneous catalysis. There are several points to be considered for optimal realization of nanoparticle/support system: high electrical conductivity for efficient electron transport, high chemical and electrochemical stability for use at various pH and electrochemical potentials, strong interactions between the support and catalyst, and efficient mass transfer. Herein, we summarize typical strategies to address surfactant issues (ultraviolet‐ozone (UVO) treatment, thermal, chemical, and electrochemical cleaning processes) as shown in **Figure** **14** and utilization of support materials (use of stable supports, nanoparticle‐support interaction, and encapsulation) and discuss future‐scope.

### 3.1 Removal of Surface Capping Agents

#### 3.1.1 UVO Treatment

UVO treatment is an effective strategy to remove various contaminants from the surface of nanoparticles. This strategy utilizes a combination of UV light and ozone, which can oxidize carbon‐containing organics into gaseous carbon dioxide. Sum frequency generation spectroscopy was used to confirm that tetradecyltrimethylammonium bromide surfactant molecules on platinum nanoparticles were removed after 15 min of UVO treatment.^[^ ^293^ ^]^ After the treatment, the shape and size of the nanoparticles were preserved, confirming the effectiveness of this treatment. This approach has been successfully extended to various nanoparticles, including Pt,^[^ ^294^ ^]^ Au,^[^ ^295^, ^296^ ^]^ Pd,^[^ ^297^ ^]^ and PtFe^[^ ^298^ ^]^ nanoparticles coated with various organic ligands such as oleylamine,^[^ ^299^ ^]^ dodecanethiol,^[^ ^296^ ^]^ and dioctadecyldimethylammonium ligands.^[^ ^295^ ^]^

#### 3.1.2 Thermal Annealing

Thermal annealing is commonly applied to remove ligands by decomposition or desorption at appropriate temperatures. The annealing conditions should be carefully chosen to prevent agglomeration or sintering of nanoparticles and preserve the original size and morphology, which are detrimental to the catalytic activity.^[^ ^300^, ^301^, ^302^ ^]^ Stamenkovic and co‐workers compared the effectiveness of thermal annealing with an acid treatment and UVO treatment on surfactant removal.^[^ ^15^ ^]^ Among them, the catalyst annealed at 185 °C showed a more exposed electrochemical surface than the others, which was analyzed by hydrogen underpotential deposition (H_upd_). Thermal gravimetric analysis confirmed that most of the surfactant was removed (with ≈45% weight loss) after 5 h of thermal annealing at 185 °C, leaving only 1% loss when the temperature was further increased to 900 °C. The electrochemical surface area (H_upd_) showed the following trend: thermally annealed > acid‐treated > UV‐ozone treated > untreated catalysts. Not surprisingly, the ORR activity was highly affected by the surfactant removal protocols, highlighting the importance of proper removal of surfactants for electrochemical performance.

#### 3.1.3 Chemical Treatment

Chemical treatment is used to remove the organic surfactants. Acetic acid is a widely used chemical for the removal of various organic surfactants at 60–90 °C.^[^ ^303^, ^304^, ^305^, ^306^ ^]^ Unfortunately, the method is insufficient to remove the ligands with strong binding, such as phosphines. Recently, ligand exchange of surfactants with an N‐heterocyclic carbene (NHC) ligand (with strong *σ*‐donors and *π*‐acceptors) was investigated as an efficient way to remove surfactants after mild acid treatment.^[^ ^307^ ^]^ As NHC is a stronger electron‐donor than phosphines, they readily replace phosphine ligands. The vibrational bands of NHC‐bound Pt, Pd, and Au confirmed the presence of oxidized NHC, indicating that the phosphine ligands were fully exchanged with NHC. The NHC ligands were subsequently removed by acetic acid treatment without heating. After treatment with acetic acid and washing, the diffuse reflectance infrared Fourier transform spectra of the samples showed no or tiny signs of organics on the surface; however, the initial sizes and morphologies of the noble metal nanoparticles (Pt, Pd, and Au) were well preserved.

#### 3.1.4 Electrochemical Method

Electrochemical strategies have also been applied to remove various ligands from the surfaces of nanoparticles.^[^ ^308^, ^309^, ^310^, ^311^ ^]^ Recently, Fan and co‐workers reported a facile and universal electrochemical method to eliminate organic surfactants.^[^ ^312^ ^]]^ They applied the following steps: 1) removal of surfactants by forming strong O or H bonds on the metal surface (M) by electrochemical reactions, and 2) recovering the initial metallic state by reduction or oxidation to remove O and H. In detail, Pt tends to be oxidized to PtO to form Pt—O covalent bond over 1.0 V (vs reversible hydrogen electrode (RHE)). Once Pt is oxidized to PtO, oleylamine can no longer coordinate with Pt because of the robust covalent bonding between Pt and O. The PtO is reduced to Pt, as the potential decreases below 0.7 V (vs RHE). Thus, the desorbed oleylamine cannot reabsorb onto the Pt surface because of its low concentration in the electrolyte due to its low solubility in aqueous solution. In turn, the hydrogen evolution reaction removes the oleylamine ligands on the nanoparticles by strong M—H bonds in the same manner as the M—O bond. In the hydrogen evolution region, M–oleylamine bonds are substituted by the M—H bond because the M—H bond is stronger than M–oleylamine. As the hydrogen desorption potential goes beyond 0.3 V (vs RHE), a clean M surface is exposed, which can then be utilized for desired catalytic reactions.

#### 3.1.5 Outlook

Various methods have been successfully used to remove surfactants. Furthermore, various analytical techniques have been utilized to prove the removal of surfactants; however, there is still a lack of careful consideration of how the physicochemical properties of nanoparticles change during the removal processes. Owing to the high surface energy of the nanoparticle surface, they may easily be reconstructed during the treatments and lose their designed functions. Therefore, monitoring the structural evolution during processes with reliable surface‐sensitive protocols and real‐time (in situ) monitoring is highly recommended. Finally, recent reports suggest that surfactants or organic molecules are no longer merely undesirable but can be utilized as positive promoters for certain reaction conditions to guide reaction selectivity by steric hindrance^[^ ^313^, ^314^, ^315^, ^316^ ^]^ or electronic effects.^[^ ^317^, ^318^ ^]^ Therefore, it is important to redefine the roles of surfactants on the nanoparticle surface as promotional or detrimental effects and how to control their functionalities according to the target catalytic reactions.

### 3.2 Effect of Support Materials

#### 3.2.1 Nanoparticle‐Support Interaction by the Electronic Effect

There are several requirements that support materials should satisfy to achieve ideal catalytic performance. First, the high electrical conductivity of the supports provides a complete electrical network for high‐rate electrochemical reactions. Second, supports should be (electro)chemically stable against corrosive environments in electrolytes as nanoparticles rely largely on supports. Third, strong interactions between nanoparticles and supports are highly recommended for the stabilization of nanoparticles. Finally, support materials with a high surface area allow nanoparticles to be adsorbed on them with proper interparticle distances. Considering the above criteria, high‐surface‐area carbon materials, including microporous carbons, carbon nanotubes, and graphene, are commonly utilized as supports for the application of nanoparticles in various electrochemical reactions. Although the oxidation of carbon can occur over 0.207 V (vs RHE) to produce CO_2_, as follows

(1) $$C+2H_{2}O\to CO_{2}+4H^{+}+4e^{-}$$

It does not significantly affect the electrochemical reactions that occur below 1.5 V (vs RHE) because the kinetics of carbon corrosion are not very fast.^[^ ^319^ ^]^ Therefore, carbon‐based materials are generally considered as ideal support materials with high electrical conductivity and (electro)chemical stability.

**Figure 15:**
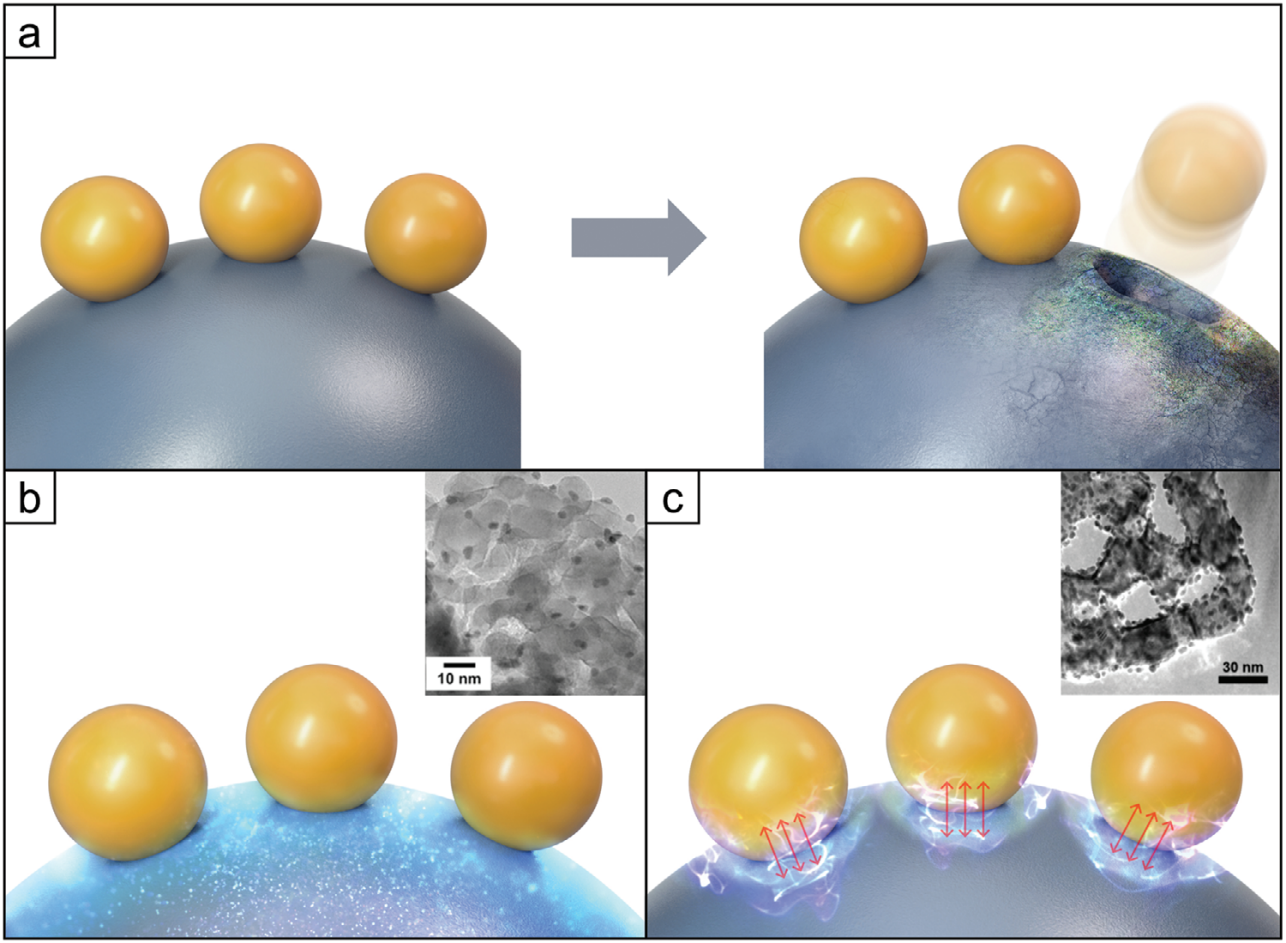
Carbon corrosion issues for conventional nanoparticle/support systems and recent approaches to overcome the problems. a) Carbon corrosion followed by nanoparticle detachment. b) (Electro)chemically inert metal oxide supports. Inset: TEM image of Pt dispersed on Nb‐doped TiO_2_. Reproduced with permission^[^ ^329^ ^]^ Copyright 2016, Elsevier. c) Nanoparticle‐support interaction to improve activity and stability. Inset: TEM image of Pt dispersed on porous TiN. Reproduced with permission.^[^ ^359^ ^]^ Copyright 2016, American Chemical Society.

However, under realistic operating conditions in devices such as start‐up/shutdown events in fuel cells and high‐potential‐based applications such as water oxidation (oxygen evolution reaction), carbon‐based supports suffer degradation by particle detachment and alteration of pore structure, resulting in performance losses,^[^ ^320^ ^]^ making them no longer an ideal platform for nanoparticle utilization (**Figure** **15**a).^[^ ^321^, ^322^ ^]^ Furthermore, the interaction between nanoparticles and carbon supports is not strong enough to stabilize nanoparticles, despite the limited discussion on this issue.^[^ ^323^, ^324^ ^]^ Metal oxides are often suggested as alternatives to overcome the carbon‐based support limitations^[^ ^325^, ^326^ ^]^ as metal oxides are generally stable in electrochemical operation windows. However, the number of possible oxide candidates is limited owing to the issue of electrical conductivity.^[^ ^327^ ^]^ One typical example is titanium‐based oxide materials. While the electrical conductivity of bare TiO_2_ is not high enough to fully support electrochemical reactions, various dopants including Ru, Mo, Ta, V, Cr, and Nb could significantly increase the conductivity that made the doped‐TiO_2_ highly applicable to electrochemical applications (Figure 15b).^[^ ^328^, ^329^, ^330^ ^]^ For example, nanostructured Ti_0.7_Mo_0.3_O_2_ was utilized as a superior support material for ORR.^[^ ^328^ ^]^ Compared to Pt/C, Pt deposited on Ti_0.7_Mo_0.3_O_2_ was more stable due not only to the stability of the support materials itself but also to the strong interaction between nanoparticles and support by electronic effect, the so‐called strong metal‐support interaction. Similar to TiO_2_, SnO_2_‐based materials have also been highlighted as potential alternatives.^[^ ^331^, ^332^, ^333^ ^]^ By doping with In, Sb, or F atoms into SnO_2_, the obtained indium‐doped tin oxide, antimony‐doped tin oxide (ATO), and fluorine‐doped tin oxide were utilized under various electrochemical conditions with high chemical stability and electrical conductivity. However, recent in situ studies on electrochemical stability revealed that tin oxide‐based materials are also vulnerable to dissolution in a broad electrochemical potential window,^[^ ^334^ ^]^ raising questions about whether oxides can be “actual” alternatives as stable support materials for electrochemical applications.

Not surprisingly, metal oxide‐based support materials are highly accentuated in oxygen evolution reaction. As we discussed already, carbon corrosion is an extremely severe problem in the high potential region (>1.5 V vs RHE). Especially, the surface oxidation of carbon followed by passivation can abruptly prohibit catalyst utilization.^[^ ^335^ ^]^ Therefore, carbon‐based supports are no longer considered in OER applications. The Strasser group successfully introduced and validated the importance of durable support materials using SnO_2_, doped with various elements (Sb, In, and F).^[^ ^336^, ^337^, ^338^ ^]^ Among the candidates, mesoporous ATO showed superior physicochemical properties such as high surface area, high electrical conductivity, and superior electrochemical stability. During the potential cycling (10 000 cycles with 500 mV s^−1^), the carbon support (Vulcan) lost ≈ 50% of its surface capacitance while ATO showed negligible capacitance loss.^[^ ^337^ ^]^ Furthermore, the superiority of the ATO support was further demonstrated when it was used to support Ir nanoparticles. Not only was the support itself stable, but also were the Ir nanoparticles effectively stabilized without a size growth due to the strong electronic effect of the support on the active metal catalyst. This system has been further improved by various metal‐support combinations with mechanistic studies,^[^ ^339^ ^]^ morphology controls,^[^ ^340^ ^]^ surface treatments,^[^ ^341^, ^342^ ^]^ and selection of various oxides.^[^ ^343^, ^344^, ^345^ ^]^

In addition to the stabilization effect on active metal nanoparticles, there have been many reports on the promotive effects of metal oxide supports in the increased reaction kinetics, such as in methanol oxidation reaction (MOR).^[^ ^346^ ^]^ Various multimetallic catalysts showed enhanced performance in combination with different oxide supports, which has been explained by two main reasons.^[^ ^347^, ^348^, ^349^, ^350^, ^351^, ^352^ ^]^ First, OH groups on the oxide surface facilitate the removal of CO_ad_ on the metal sites via bifunctional mechanism and increase MOR activity, because CO_ad_ (reaction intermediate of MOR) is strongly adsorbed and blocks (poison) the active surface of metal nanoparticles. Second, the electronic effect between metal nanoparticles and metal oxide supports improves charge transfer between them. In ethanol oxidation reaction (EOR), the dissociation of C—C bond has been a major challenge to oxidize ethanol completely to CO_2_, prohibiting further development of alcohol‐based direct fuel cells. To solve this issue, the Adzic group designed a SnO_2_‐supported multimetallic PtRh system as a synergistic EOR catalyst.^[^ ^353^ ^]^ SnO_2_ strongly adsorbs water and provides OH species to oxidize CO on Rh sites while Pt facilitates the ethanol dehydration. Furthermore, SnO_2_ also modifies the electronic state of Rh nanoparticles to afford optimum binding of intermediate species, enhancing the overall reaction kinetics.^[^ ^353^ ^]^ In order to complement the limited range of multimetallic nanoparticle design, many combinations of metal‐support structures have been studied, tuning the compositions^[^ ^354^, ^355^ ^]^ and metal‐support interfaces.^[^ ^356^, ^357^, ^358^ ^]^

In contrast to oxide‐based systems, nitrides and carbides have expanded their applicability owing to their high electrical conductivity, among which TiN has received much attention due to the higher conductivity (40 kS cm^−1^ in bulk phase).^[^ ^359^ ^]^ TiN supports with various morphologies, including particulate, tubular, hollow, and 3D structures, were found to stabilize noble metal‐based catalysts better than carbons (Figure 15c).^[^ ^359^, ^360^, ^361^ ^]^ Furthermore, the introduction of Nb, Cr, or Ni into TiN has proved to provide more active and stable electrochemical performance.^[^ ^362^, ^363^, ^364^ ^]^ Despite the promising aspects of their applicability, the issue of surface oxidation continues to be pointed out as the primary limitation of nitride‐based supports. In addition, most studies based on nitrides are limited to half‐cell level tests, so whether they can be applied to actual devices, such as membrane electrode assembly, is an important remaining challenge. On the other hand, light element‐based carbides and nitrides, such as hexagonal boron nitride and boron carbide (BC), have recently been studied to have strong electronic interactions with catalysts having long‐term durability.^[^ ^365^, ^366^ ^]^ In Pt/BC systems, strong dipole interactions between metal nanoparticles and supports reduced the mobility of Pt nanoparticles, thus mitigating agglomeration. Furthermore, carbide‐supported nanoparticles are considered to be slightly less prone to form oxide surfaces, which is directly linked to the mitigation of the anodic and cathodic Pt dissolution during electrochemical potential cycles.^[^ ^366^ ^]^

#### 3.2.2 Nanoparticle‐Support Interaction by the Geometric Effect

**Figure 16:**
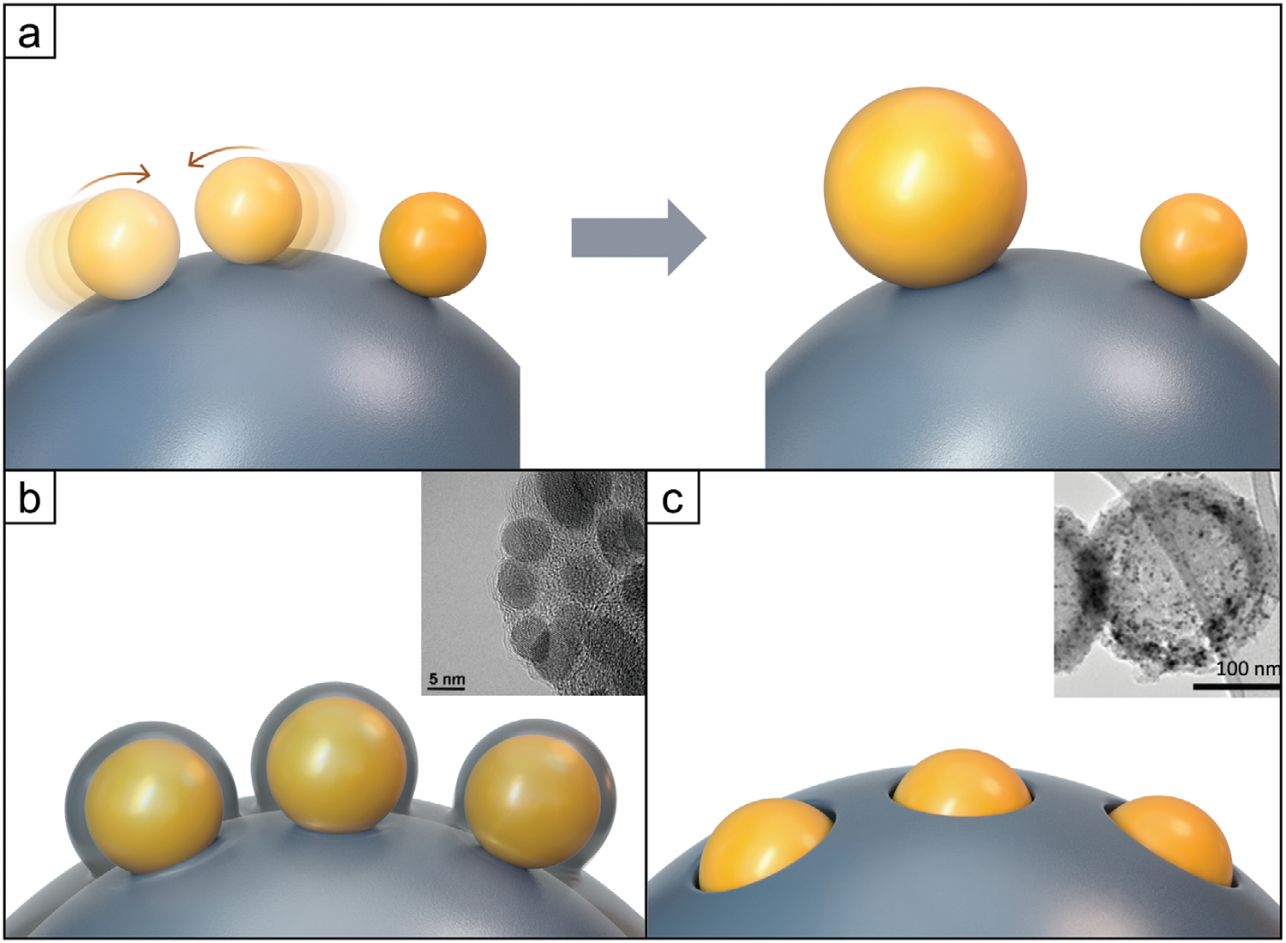
Nanoparticle degradation mechanism originated from migration of nanoparticles on support and recent approaches to overcome the problem. a) Nanoparticle agglomeration via weak interaction with support. b) Geometrical encapsulation method to stabilize nanoparticles. Inset: TEM image of carbon‐shell‐encapsulated PtFe nanoparticles. Reproduced with permission.^[^ ^134^ ^]^ Copyright 2015, American Chemical Society. c) Geometric confinement in porous structures. Inset: TEM image of geometrically pore‐confined Pt_3_Ni‐Mo octahedra. Reproduced with permission.^[^ ^386^ ^]^ Copyright 2018, American Chemical Society.

Simple physical attachment is a conventional approach to stabilize nanoparticles on supports. As one of the major degradation mechanisms of nanoparticles is particle–particle agglomeration caused by weak interaction between them with supports (**Figure** **16**a), various efforts have been devoted to enhancing the interaction by heteroatom doping, strong dipole interaction, and functional groups.^[^ ^366^, ^367^, ^368^, ^369^ ^]^ Recently, two different approaches have been widely adopted to overcome the instability of catalysts on supports: 1) geometric confinement of nanoparticles via encapsulation with stable overlayers (Figure 16b) and 2) geometric confinement of nanoparticles in nanoscale pore structures to mitigate their migration (Figure 16c). First, encapsulation of nanoparticles is usually performed by confinement in carbon nanotubes or the formation of carbon overlayers.^[^ ^134^, ^370^, ^371^, ^372^ ^]^ Particularly, the use of a very thin carbon layer is attracting attention in terms of being able to stabilize nanoparticles while maintaining mass transfer effectively.^[^ ^134^ ^]^ When the protection layer is too thick, it is difficult for the reactants to penetrate, while nanoparticles cannot be protected if the layer is too thin. Therefore, it is important to control and balance the thickness of the protection layer to simultaneously determine the optimal conditions for achieving high activity and stability at the same time. This strategy has been successfully extended to various types of nanoparticles (metal oxides, nitrides, and phosphides)^[^ ^373^, ^374^, ^375^, ^376^ ^]^ and different carbon layer formation strategies have been proposed.^[^ ^377^, ^378^, ^379^, ^380^ ^]^ Second, geometric confinement in porous structures can also stabilize nanoparticles against migration on supports and detachment from the supports.^[^ ^381^, ^382^, ^383^ ^]^ Using porous structures, nanoparticles are geometrically confined in pore space and maintained their size during electrochemical measurements without agglomeration and particle detachment, minimizing activity losses.^[^ ^384^, ^385^, ^386^, ^387^ ^]^ Recently, encapsulation of nanoparticles in porous structures was further extended to the preconfinement of nanoparticles in porous matrices such as metal organic frameworks.^[^ ^388^, ^389^ ^]^ After encapsulation of nanoparticles in a porous frame, the thermal annealing process can confine nanoparticles in porous carbon supports. Furthermore, the surface of nanoparticles can be wrapped with very thin‐layer carbon upon annealing, achieving a dual stabilization step with thin carbon‐layer encapsulation and geometric confinement in the porous structure. In addition, attempts to simultaneously achieve nanoparticle synthesis and stabilization effects by introducing metal precursors to porous carbons or frameworks have been recently reported. Moreover, highly advanced nanoparticle design strategies for their stable utilization have been continuously proposed to overcome the limitations of conventional nanoparticle/support systems.^[^ ^182^, ^390^, ^391^ ^]^

## 4 Summary and Perspectives

NMMNs have been an indispensable part of active research in electrocatalysis because of their intrinsic electronic properties that give close‐to‐optimal binding energies of reacting species, such as reaction intermediates. However, as the demand for higher electrocatalytic performance has increased, researchers have developed different approaches and strategies to produce desired or novel multimetallic nanostructures based on noble metals. Considering the myriad of studies done so far on the synthesis of NMMNs, although we still have far more to go, we attempted to provide a comprehensive but concise review of the controlled synthesis of NMMNs after classifying their structures into random alloys, single‐atom alloys, high‐entropy alloys, ordered‐intermetallics, core–shell structures, and other heterostructures.

The current status of each structure since last two decades can be summarized as follows. 1) For random alloys, control of size and composition relies primarily on reaction kinetics that affects the nucleation and growth of nanoparticles, while recent state‐of‐the‐art catalysts take advantage of novel shapes that can be tuned by facet control (including HIFs), etching, galvanic replacement, or the Kirkendall effect. 2) As subgroups of alloys, single‐atom alloys, and high‐entropy alloys have unusual structural features, while controlled synthetic methods are still underdeveloped. The key points to obtain SAA and HEA are the effective deposition of only one separate atom and fast simultaneous nucleation of multiple metal elements, respectively. 3) Although various intermetallic phases of Pt, Pd, and Au have been constructed as nanoparticles, precise control over the size and composition is still limited to a few metal combinations, calling for modified or novel synthetic strategies. 4) Core–shell nanoparticles have been effectively prepared by seed‐mediated routes in a controlled manner, while one‐pot synthesis and transformation of alloys into core–shell nanoparticles have also been adopted as alternatives. 5) Other heterostructured NMMNs have also been prepared by seed‐mediated approaches, revealing the important role of surfactants in the selective anisotropic deposition of secondary metal species by their nonuniform binding on different surface sites of seeds. Additionally, the lattice mismatch and immiscibility between metal elements also contribute to the formation of heterostructures.

In addition to the discussed issues regarding the designed synthesis of various kinds of NMMNs, their subsequent translation into the active form of electrocatalysts should be viewed as equally important. Although the presence of surfactants plays a vital role in controlling the size and shape of NMMNs, they should generally be removed to expose the active metal surfaces to achieve desirable performance fully. Representative surface treatments were introduced in this review, among which physical treatments using ultraviolet ozone or thermal annealing have been widely used. One the other hand, careful selection and modification of the support materials are required to enhance the long‐term stability of NMMNs. Protection of active nanoparticles is achieved by surface encapsulation (usually by thin carbon) or geometric confinement of each particle in the pores of the supports.

Furthermore, future research should focus on overcoming the following challenges in the preparation of active, selective and durable electrocatalysts. First, controlled synthesis of SAAs and HEAs with carefully tuned active sites can provide well‐defined catalytic active sites (by SAAs) or a wide range of surface atomic ensembles (by HEAs) for an unprecedented activity and selectivity in a target reaction. Development of more generalized and reliable synthetic strategies would stimulate in‐depth investigations in either empirical or theoretical ways. Secondly, synthesis of uniformly sized, more atomically ordered and shape‐controlled intermetallic nanoparticles may resolve the current stability issues of NMMN‐based electrocatalysts that impede their practical applications. Efforts to discover and analyze intermetallic nanomaterials of unexplored compositions would expand our options to find the most suitable practical catalyst. Third, monitoring the structural evolution of NMMNs upon surfactant removal would enable a precise activation of as‐synthesized surface active sites, preventing their unwanted deformation into less active forms. In situ microscopic and spectroscopic techniques are highly promising for the detailed observation of structural changes during surfactant removal steps. Fourthly, systematic comparison of the effect of support structures on the activity and stability of NMMNs is still limited, which becomes more important when NMMNs are applied for practical devices. Finally, cost‐effective, large‐scale production of supported NMMN‐based catalysts is indispensable for their industrial‐level uses but scaling up the synthesis usually compromises their unique structural properties and performances. Thus, novel or modified synthetic methods are to be developed to obtain active and durable supported NMMNs in a gram‐scale or a kilogram‐scale.

## Conflict of Interest

The authors declare no conflict of interest.
